# An analytical framework for decoding cell type-specific genetic variation of gene regulation

**DOI:** 10.1038/s41467-023-39538-7

**Published:** 2023-06-30

**Authors:** Yanyu Xiao, Jingjing Wang, Jiaqi Li, Peijing Zhang, Jingyu Li, Yincong Zhou, Qing Zhou, Ming Chen, Xin Sheng, Zhihong Liu, Xiaoping Han, Guoji Guo

**Affiliations:** 1 Center for Stem Cell and Regenerative Medicine, and Bone Marrow Transplantation Center of the First Affiliated Hospital, Zhejiang University School of Medicine, Hangzhou, Zhejiang 310000 China; 2 Liangzhu Laboratory, Zhejiang University Medical Center, Hangzhou, Zhejiang 311121 China; 3 College of Life Sciences, Zhejiang University, Hangzhou, Zhejiang 310003 China; 4 Life Sciences Institute, Zhejiang University, Hang Zhou, Zhejiang 310058 China; 5 Zhejiang Provincial Key Lab for Tissue Engineering and Regenerative Medicine, Dr. Li Dak Sum & Yip Yio Chin Center for Stem Cell and Regenerative Medicine, Hangzhou, Zhejiang 310058 China; 6 Zhejiang University-University of Edinburgh Institute, Zhejiang University School of Medicine, Zhejiang University, Hangzhou, 314400 China

**Keywords:** Computational biology and bioinformatics, Cell biology, Gene regulation

## Abstract

A deeper understanding of genetic regulation and functional mechanisms underlying genetic associations with complex traits and diseases is impeded by cellular heterogeneity and linkage disequilibrium. To address these limits, we introduce Huatuo, a framework to decode genetic variation of gene regulation at cell type and single-nucleotide resolutions by integrating deep-learning-based variant predictions with population-based association analyses. We apply Huatuo to generate a comprehensive cell type-specific genetic variation landscape across human tissues and further evaluate their potential roles in complex diseases and traits. Finally, we show that Huatuo’s inferences permit prioritizations of driver cell types associated with complex traits and diseases and allow for systematic insights into the mechanisms of phenotype-causal genetic variation.

## Abstract (web summary)

Understanding genetic regulation and the underlying mechanism for genetic associations with human phenotypes is complicated by cellular heterogeneity and linkage disequilibrium. Here, the authors propose an analytical framework to decode genetic variation of gene regulation at both cell-type and single-nucleotide levels.

## Introduction

The majority of variants associated with human genetic disorders lie outside of gene coding regions and there has been no unified method to decode their impact^1^. Despite that expression quantitative trait loci (eQTLs) have greatly facilitated functional interpretations of genome-wide association study (GWAS) findings, a deeper understanding of the underlying biological mechanisms has been limited by heterogeneous cellular compositions in bulk tissues. Several studies^2–5^ have identified high degree of cell type specificity of gene regulation by integrating single cell RNA-sequencing (scRNA-seq) and population genetics from the same cohort of donors. These works provide an important methodological framework to reveal cell type-specific eQTLs that are unobservable with bulk RNA-seq. However, scRNA-seq and genetic profiles from a large number of individuals are often difficult to obtain, making it intractable to evaluate the genetic regulatory effects in all cell types.

To date, many large projects such as Genotype-Tissue Expression (GTEx) consortium^6^ have devoted major efforts to identify eQTLs by measuring the averaged RNA signals from all cell types in a bulk sample. Meanwhile, cell landscapes of major human tissues based on single-cell transcriptome profiles have been generated to characterize gene expression signatures in cell populations^7^. In addition, ab initio predictions of variant effects based on DNA sequences have been achieved with convolutional neural network (CNN) models^8–12^ through modeling regulatory and epigenomic elements over the last decade. These advances provide an unprecedented opportunity to break through current limitations and explore genetic regulation at higher resolutions and larger scales.

In this study, we sought to utilize the existing bulk-level data resources and the single cell-level cell landscapes to uncover the cell type-specific effects of genetic variation on gene expression. By exploiting the single-cell gene expression profiles, we extend the existing method of sequence-based variant predictions and shed light on cell type-dependent cis-regulatory loci by investigating the interaction effects between genotypes and estimated cell type proportions in tissue samples with a linear regression model. This pipeline enables analysis of genetic regulation at the cell type level using available bulk-level datasets and eventually formalizes the analytical framework of Huatuo. We demonstrate that Huatuo enables a higher resolution exploration of genetic regulation, and then apply it to generate a comprehensive landscape of cell type-specific genetic variation. Finally, we extend the landscape to provide systematic insights into complex traits and diseases, including potential driver cell types and functional mechanisms of trait-causal and disease-causal genetic variation.

## Results

### Building a framework to decode cell type-level and landscape-scale genetic variation of gene regulation

**Fig. 1:**
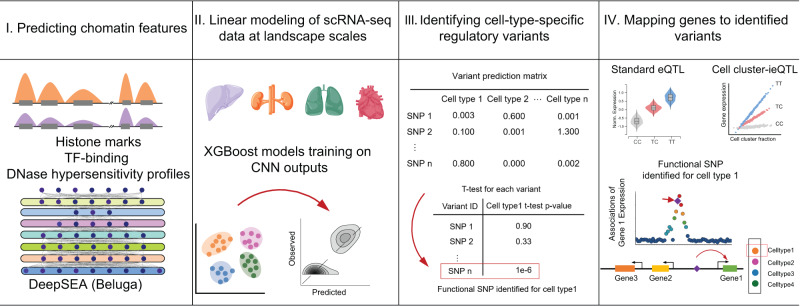
Overview of Huatuo workflow. The framework employs a deep-learning-based model to predict chromatin factor features around the genome regions centered on transcription start sites (TSSs), then extends the sequence-based chromatin effect predictions to cell type levels by modeling landscape-scape single-cell gene expression profiles using XGBoost-based linear regression. All the predicted variants are ranked according to Student’s t-tests to acquire the variant sets with specific functional regulatory effects for target cell types. Finally, Huatuo combines the genetic associations with gene expression levels, including both standard eQTLs and cell cluster-ieQTLs, to infer genes that can be perturbed by the putative functional genetic variation.

Exploring the effect of non-coding variants is complicated by genomic sequences, heterogeneous chromatin configurations, and structural interactions between regulatory elements. Here we propose an analytical framework, named Huatuo, to unravel the causal mechanisms underlying genetic variation of gene regulation. By applying the Huatuo framework to single-cell transcriptome profiles, we can perform a genome-wide analysis to investigate genetic regulation at cell type and single-nucleotide resolutions. The Huatuo framework consists of four main stages (Fig. 1 and Methods). In brief, we first employ a publicly available CNN model^8^ to integrate information from a wide sequence context, around the ±20 kb genome region centered on transcription start site (TSS) of each gene (Supplementary Data 1). Next, to capture the chromatin configuration in specific cell types, we fit the gene expression derived from scRNA-seq data with XGBoost regression models^13^ using the integrated sequence information. Both of the used sequence and regression model are found to be suited for this application in the benchmarks testing downstream ability to predict gene expression levels (Supplementary Fig. 1). Then, by comparing the CNN predictions transformed by the fitted cell cluster models between the reference and alternative allele sequences, chromatin-level effects of in silico mutagenesis in the cell types of interest can be estimated. We consider the nucleotide substitutions with significantly higher effects from de novo predictions in the focal cell type as cell type-specific functional regulatory variants. To shed light on roles of the putative functional variants in regulating expression of specific genes, we finally perform an integration analysis with population-based associations, including both the standard eQTLs mapped directly in bulk tissues and the interaction expression quantitative trait loci (ieQTLs) estimated with scRNA-seq profiles. The integration with eQTLs and ieQTLs enables a plausible inference of associations between the putative regulatory variants and genes, including both proximal and long-range interactions. The proposed framework takes advantage of the current state-of-the-art predictions to disentangle causality for the complicated genetic regulation, and seeks independent statistical support for the ab initio variant predictions from population-based association calculations.

In this study, we applied Huatuo to the single-cell transcriptome profiles from the Human Cell Landscape (HCL)^7^. We built predictive models for each of the 357 HCL cell clusters, mapped ieQTLs for multiple cell clusters using the GTEx datasets^6^ and performed integration analysis by combining de novo variant predictions with population-based associations (Supplementary Figs. 2–4 and Methods).

### Performance evaluation of ab initio variant predictions

**Fig. 2:**
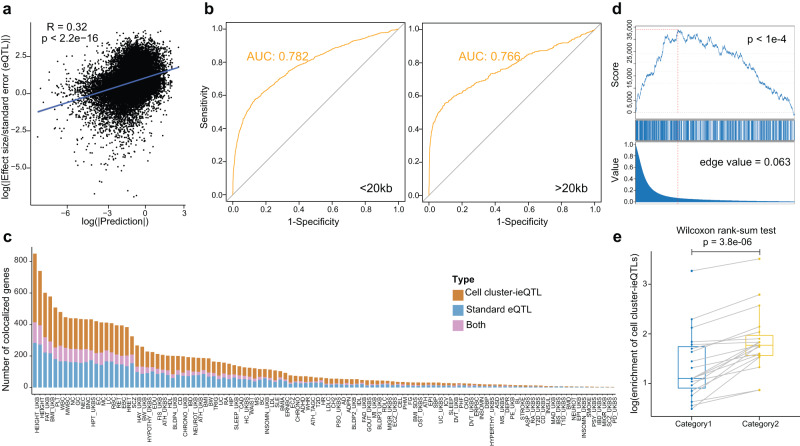
Benchmarking de novo variant predictions and cell cluster-ieQTLs in Huatuo. **a** Dot plot revealing a highly significant correlation between predicted variant effects and eQTL summary statistics. The rho and *p*-value were derived from Spearman rank correlation test, two-sided. **b** ROC curves derived from logistical classifiers trained to predict fine-mapped eQTLs located within 20 kb (left) or more than 20 kb away (right) from TSSs. **c** Bayesian colocalization uncovering a large number of colocalized signals between GWASs and cell cluster-ieQTLs that are not observed with standard eQTLs. The y-axis represents 114 GWASs, and the *x*-axis represents the number of colocalized genes between GWAS polygenetic associations and standard eQTLs or cell cluster-ieQTLs. Bar colors indicate the colocalized positive signals (PP4 > 0.85) for either only standard eQTLs (light blue), or only cell cluster-ieQTLs (orange), or both (pink). GWAS abbreviation: Supplementary Data 3. **d** GSEA plot showing the enrichment of curated gene-trait pairs in colocalized signals between GWASs and cell cluster-ieQTLs. The *p*-value was estimated by permutation (one-sided, 10,000 permutations). **e** Boxplot showing the enrichment of cell cluster-ieQTLs for two categories of putative regulatory variants identified by cell cluster models. Category 2 refers to the putative regulatory variants for which the absolute values of estimated effects were notably greater in the predictions from cell cluster models compared with corresponding tissue-level models, and category 1 represents the remaining putative regulatory variants. Each dot represents one tested tissue. Statistical test: one-sided Wilcoxon rank sum test.

To benchmark de novo variant predictions from the Huatuo framework, we first tested whether the predicted effects could reproduce the results of population-based association analysis based only on DNA sequences. Predictions from all the models in each tissue were pooled together and compared to the standard eQTLs from GTEx consortium^6^. We found that the highest absolute value of eQTL summary statistics (effect size/standard error) and predicted variant effects within a linkage disequilibrium (LD) block exhibited significant correlation (median Spearman R = 0.30 across tested tissues, Figs. 2a, r2 > 0.6). Then, we examined whether the variant predictions could pinpoint the causal variants for eQTL association signals by testing on a published dataset of fine-mapped eQTLs^14^, using the highest prediction per variant in one tested tissue. Huatuo could distinguish the positive fine-mapped variants for a gene from the negative ones in the same LD block region with a mean AUROC of 0.780. Importantly, the deep-learning-based discrimination of putative causal eQTLs displayed good performance for both the distal and proximal regulatory regions (Fig. 2b). Additional evidence for the ability of Huatuo to pinpoint putative causal functional variants came from the identification of pathogenic mutations using the variant prediction model trained for HUDEP-2 cells. The predicted effects of known pathogenic variants^15^ located in the regulatory region of γ-globin (HBG) gene were 3.25-fold stronger than those of randomly selected variants with matched distance to the transcription start site (TSS) around the gene (95% confidence interval [CI], 1.62–6.16).

### Mapping cell cluster-ieQTLs to provide more insights into genetic regulatory effects in bulk tissue samples

Variant regulatory effects in standard eQTLs can be obscured by the heterogeneous cellular compositions in bulk tissues, especially for those derived from rare cell types in the samples. In Huatuo, cell cluster-ieQTLs are mapped to provide more insights into the genetic regulatory effects in bulk tissue samples (Methods). We define cell cluster-ieQTLs as the interaction expression quantitative trait loci that modify gene expression levels with a cell cluster-dependent manner (Supplementary Fig. 3a). For instance, in the arterial tissue samples from GTEx individuals with a higher G allele dosage (rs35228013), the expression level of *ZC3H3* was negatively correlated with the estimated cell cluster proportion of B cells. In addition, the association between the SNP genotype and the gene expression was stronger in the tissue samples with a higher estimated cell cluster proportion of B cells (Supplementary Fig. 3b). This observation indicates that allele dosage effects on gene expression can be heterogeneous between cell types.

To validate cell cluster-ieQTLs mapped in the Huatuo framework, we compared the yielded results (Supplementary Data 2) with a previous ieQTL study^16^. Different from standard eQTLs that are likely to be conserved across cell types, the ieQTL test only focus on the regulatory effects dependent on specific cell types. In agreement with this, the cell cluster-ieQTLs showed significantly more overlap with the reported ieQTLs compared to standard eQTLs (median odds ratio = 3.769 across tissues, Fisher’s two-sided exact test). To evaluate the ability of cell cluster-ieQTLs to reveal variant-gene associations, we performed Bayesian colocalization between cell cluster-ieQTLs and 114 publicly available GWAS datasets using the coloc package^17^ (Supplementary Data 3), and then employed a “silver standard” dataset^18^ to examine the colocalized results. This colocalization analysis revealed a large number of colocalized signals not observed with standard eQTLs, which showed significant enrichment for the gene-trait in the “silver standard” dataset (posterior probability of colocalization PP.H4 > 0.85, Fig. 2c, d, Supplementary Fig. 5 and Supplementary Data 4).

It is noteworthy that ieQTLs can originate from the tested cell types, but other cell types with proportion estimates related to the tested ones may also contribute. Due to the interdependence between cell cluster estimates, it can be challenging to determine their exact cellular origin based solely on parametric statistics of association tests. However, the de novo variant predictions in Huatuo provide a solution to characterize the cell type specificity of ieQTLs. Specifically, ieQTL SNP-gene associations are considered to be derived from the tested cell type exclusively if the ieQTL loci display a strong degree of linkage disequilibrium with putative functional variants that are also predicted to be specific to this cell type. The ieQTLs whose genotype main effects maintain a consistent sign and show a decrease from low to high estimated cellular proportions are defined as negatively correlated ieQTLs (Supplementary Fig. 3e). These ieQTLs are excluded from the identification of cell type-specific ieQTLs, as they generally capture the genetic regulatory effects that are active in other cell types.

To further validate the identified cell type-specific ieQTLs, a replication analysis was conducted using sc-eQTLs discovered in the OneK1K study^3^. We analyzed four ieQTL cell types in peripheral blood that have matched OneK1K cell types with over 600 independent sc-eQTLs identified. The replication analysis was limited to only those genes that were tested in the corresponding cell types in both studies. The results showed that, on average, 37% of ieQTL SNP-gene pairs for B cells, T cells, monocytes and NK cells were replicated in their matched OneK1K cell types. Among these cell types, T-cell ieQTLs exhibited the highest replication rate of 55.4%, likely due to the largest number of eQTLs identified for T cells in the OneK1K study. This suggests an improved replication capacity with the inclusion of larger replicating datasets. In addition, the replication analysis reflected cell specificity, with the replication rates of matched cell types consistently higher than those of other ieQTL cell types (Supplementary Table 1).

For each tissue, we found the cell cluster-ieQTLs were significantly enriched in the putative regulatory variants combined from the cell cluster predictive models (Bonferroni corrected *p*-value < 0.05 for 18/20 tissues). We further trained additional tissue-level models using expression profiles of bulk tissues and compared the ab initio predictions of variant effects between the cell type-level and the tissue-level models. We found that the cell cluster-ieQTLs skewed toward greater enrichment in the variants with significantly higher predictions from the cell cluster models (Wilcox test *p* = 3.8e−6, Fig. 2e), showing the comparability between cell cluster-ieQTLs and predictions from cell cluster models in the discovery of cell type-specific genetic regulatory effects.

### An inferred landscape of cell type-specific genetic variation of gene regulation

**Fig. 3:**
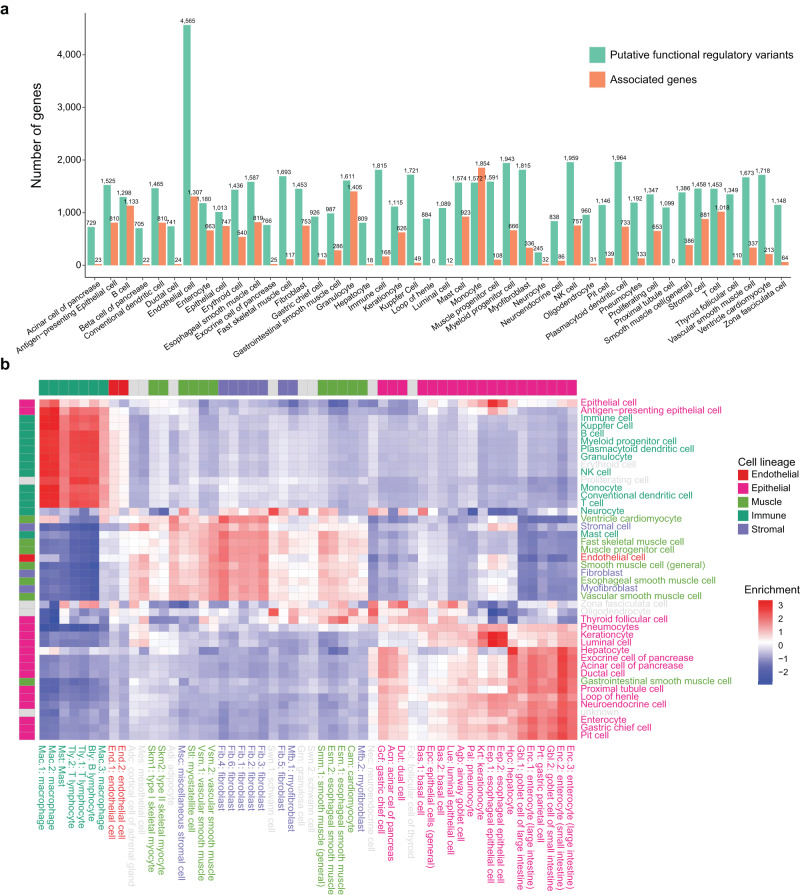
A Huatuo inferred genetic variation landscape for 44 major cell types from 20 human tissues. **a** The number of putative cell type-specific functional regulatory variants and associated genes inferred from Huatuo in each cell type. **b** Systematical validation of putative cell type-specific regulatory variants using an external scATAC-seq dataset. The heatmap shows z-scores for the enrichment of putative regulatory variants of each investigated HCL cell type (*y*-axis) in the differential chromatin accessibility of each cell type from an external scATAC-seq dataset (*x*-axis). The enrichment was evaluated using AUC scores, reflecting the extent of agreement between the inferred cell type-specific regulatory variants and the observed cell type-specific regulatory regions. The color-coding of labels on both the *x*-axis and *y*-axis indicates the cell lineage assignment of investigated cell types.

Using the Huatuo framework, we generated a comprehensive landscape of cell type-specific genetic variation for 44 major cell types from 20 human tissues. The landscape identified a total of 13,182 cell type-specific functional regulatory variants and 6181 associated genes likely to be perturbed by the putative causal regulatory variants (Fig. 3a and Supplementary Data 5). To examine whether the landscape recapitulated the cell type specificity of genetic regulation correctly, we benchmarked the 13,182 identified variants against the associated cell type-specific open chromatin regions from an external scATAC-seq dataset^19^ using an enrichment analysis (Methods). Hierarchical clustering of the enrichment results was observed to reflect a well-known hierarchical lineage relationship among these cell types. In addition, the putative regulatory variants inferred from one cell type were found to be specifically enriched in the differential open chromatin peaks of cell types from the same cell lineage, showing overall consistency between Huatuo inferred and actually measured cell type-specific regulatory regions (Fig. 3b).

Since ieQTLs are thought to be capable of providing insights into the cell type-dependent genetic regulation, we also performed a similar comparison between the cell cluster-ieQTL associations of the cell types with the open chromatin regions from scATAC-seq. By contrast, the enrichment of ieQTL SNPs exhibited less cell type specificity for the cell type-specific regulatory regions (Supplementary Fig. 6). This could be due to confounding effects from other cell types of which cellular proportion estimates in tissue samples were correlated with the focal ones, as already mentioned. Therefore, we believe that the devised Huatuo framework can benefit from the sequence-based de novo variant predictions to identify regulatory genetic variation specific for target cell types.

### Performance evaluation of Huatuo’s inferences to shed light on complex traits and diseases

We extended the Huatuo genetic variation landscape to provide insights into the mechanisms underlying the GWAS associations of complex traits and diseases. The logistic regression classifiers on the basis of Huatuo’s inferences enabled identifying the significantly trait-associated variants with a median of 0.745 AUROC scores (Supplementary Data 6), demonstrating its ability to shed light on the genetic regulation that are causally linked to a variety of human phenotypes. We further used the identified cell type-specific functional regulatory variants to partition the heritability of 114 GWAS traits and observed significant heritability enrichment, with 0.12% of SNPs explaining an average of 13.1% of SNP-based heritability^20^ (Bonferroni-corrected enrichment *p* < 0.05 in 73 GWASs, Supplementary Data 7). Besides, despite the heritability enrichment for some GWASs did not reach the significance threshold, inflation of the identified variants in the quantile-quantile (QQ) plots of the traits advocates their etiological importance (Supplementary Fig. 7). These results suggested that the putative cell type-specific genetic regulation inferred from Huatuo may generally serve as an important intermediate phenotype to affect the complex diseases and traits.

### Using Huatuo’s inferences to prioritize the cell types associated with complex traits and diseases

**Fig. 4:**
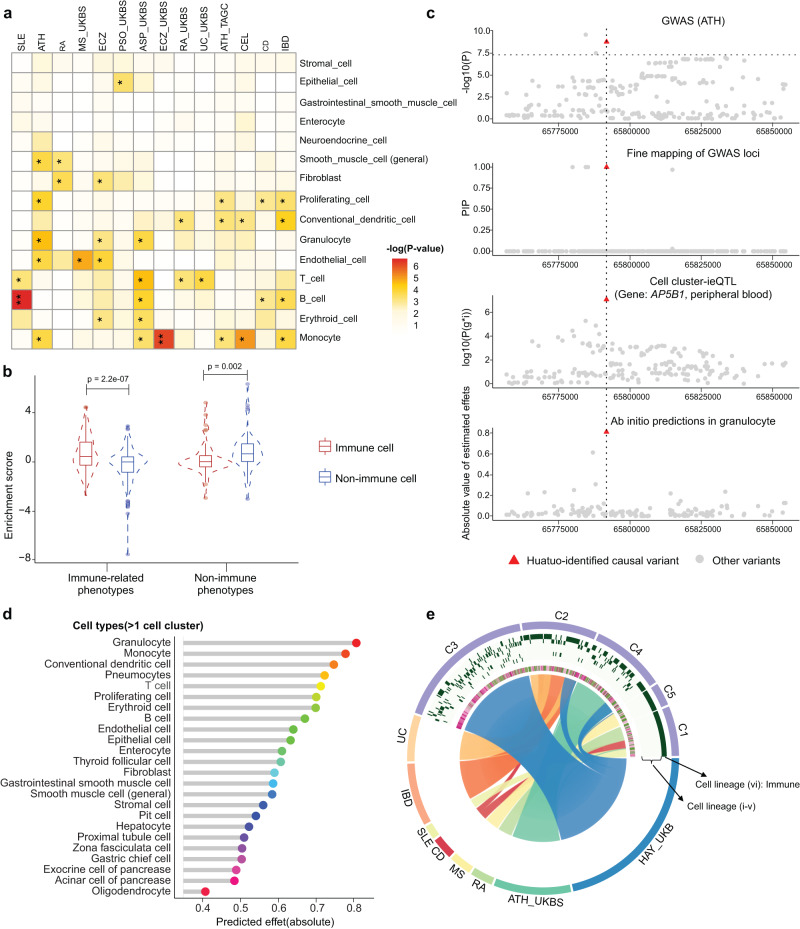
Huatuo provides cell type and variant-level insights into biological mechanisms of complex traits and diseases. **a** Association significance between prevalent cell types and immune-related traits based on stratified LD score regression analysis. The one-sided *p*-value was generated using the LDSC software. *Nominal *p* < 0.05; **Bonferroni-corrected *p* < 0.05. GWAS abbreviation: Supplementary Data 3. **b** Cell type-specific regulatory variants identified for the immune cell types (including B cells, T cells, conventional dendritic cells, erythroid cells, monocytes and neutrophils) and the nonimmune cell types (including endothelial cells, enterocytes, epithelial cells, fibroblasts, general/gastrointestinal smooth muscle cell, neuroendocrine cells and stromal cells) showing significant differences in heritability enrichment for immune-related (*n* = 23) and non-immune (*n* = 7) GWAS categories. Two-sided Wilcoxon signed rank test. Box plots depict the interquartile range (IQR), whiskers depict 1.5× IQR. **c** An example for mapping of phenotype-causal genetic variation to cell type-specific functional regulatory variants. From top to the bottom: Plots of GWAS associations of asthma. The dashed line indicates the genome-wide significance threshold; The two-sided *p*-value was obtained from the GWAS study; posterior inclusion probability (PIP) prioritized by fine mapping of the GWAS loci using SuSiE; plots of cell cluster-ieQTLs or standard eQTLs; ab initio predictions of variant effects from the cell cluster model. For all panels, the mapped functional variant is highlighted with red triangles. **d** Lollipop diagram showing the mean values of predicted effects of the mapped causal functional variant in (**c**) across cell clusters of the same cell type. Cell types with more than 1 cell cluster were presented. **e** Circos plot displaying the variant-to-function mapping for immune-related traits. Tracks from insides to outsides show cell types, corresponding cell lineages, GWAS traits and the functional regulatory variants that are unsupervised clustered (C1-C5). Cell lineages include (i) Stromal (ii) Endothelial (iii) Secretory (iv) Epithelial (v) Muscle (vi) Immune. Trait-variant links were displayed in the circos plot if the number of mapped functional variants in one cell lineage for the investigated GWAS trait exceeds 5. GWAS abbreviation: Supplementary Data 3.

We then interrogated the driver cell types associated with complex traits and diseases by performing cell type-specific stratified LD score regression^21^ using the Huatuo genetic variation landscape, estimating the marginal increases in the SNP-based heritability enrichment for each cell type (Methods). The Huatuo-based prioritization of associations between cell types and phenotypes allows recapitulating well-known mechanisms. For example, across all the tested cell types, we found the top enriched cell type for stroke^22^ and schizophrenia^23^ were vascular smooth muscle cell (*p* = 0.026) and oligodendrocyte (*p* = 0.023), respectively (Supplementary Fig. 8). Specially, we investigated the relationship between a set of immune-related phenotypes and the prevalent cell populations in human tissues. The analysis also confirmed previous findings like the associations of lupus (SLE) with B cells^24^, asthma (ATH) with granulocytes^25^ and rheumatoid arthritis (RA) with T cells^26^ (Fig. 4a). To further validate the cell type specificity of enrichment, we compared the associations between different categories of cell types and complex phenotypes. As expected, we observed a clearly stronger association of immune-related phenotypes with immune cells and non-immune phenotypes with non-immune cells (Fig. 4b). Notably, despite this, we also identified several significant associations between the immune-related phenotypes and structure cells, like multiple sclerosis (MS) with endothelial cell, psoriasis (PSO) with epithelial cell and RA with fibroblast (Fig. 4a). The observations were supported by recent advances^27^ that highlighted the roles of structure cells in the pathology of autoimmune diseases. Therefore, we believe that Huatuo can be used to facilitate novel insights in the associations between cell types and complex traits and diseases.

### Using Huatuo’s inferences to provide insights into the mechanisms of phenotype-causal genetic variation

Importantly, the Huatuo genetic variation landscape can provide systematic insights into the mechanisms of complex traits and diseases by mapping causal variants to the putative cell type-specific genetic regulation at single-nucleotide resolutions. We performed SuSiE fine-mapping analysis to acquire the 95% credible sets that were considered as the smallest variant set to have a causal variant driving the observed GWAS association signals^28^. We then tested whether our identified functional regulatory variants were exactly located in the 95% credible sets to confirm that the causal variants altering gene expression levels and affecting complex phenotypes were shared. Across the 114 GWAS datasets, the variant-to-function mapping nominated a total of 699 putative cell type-specific regulatory variants underlying the predisposition to acquiring complex traits and diseases. In addition, 672 genes correlated with the variants were reported to affect the biology relevant to the corresponding phenotypes (Supplementary Data 8 and Supplementary Fig. 9).

For example, based on the results from Huatuo, rs10791824 represents the functional variant with a specific transcriptional-regulation-disrupting effect in the cellular context of granulocytes, and shows a significant association with *AP5B1* gene expression abundance in peripheral blood. We reasoned that the allele-specific gene regulation may act as the potential mechanism leading to the genetic risk for asthma (Fig. 4c, d). Similarly, the transcriptional regulation of *LRP1* altered by rs1385526 in smooth muscle cells and *PGAP3* altered by rs10558975 in enterocytes were considered to underlie hayfever/allergic rhinitis and inflammatory bowel disease (IMD), respectively (Supplementary Fig. 10). In addition, the variant-to-function mapping can reflect cellular contribution to the genetic risk factors. The results of immune-related phenotypes exemplified this, exposing related transcriptional regulation in immune cells underlying the majority of causal variants. This analysis also revealed the genetic regulation in structure cells contributing to a fraction of phenotype-causal genetic variation, consistent with our observations from cell type heritability enrichment (Fig. 4e).

## Discussion

In this study, we introduce Huatuo, a versatile framework that enables systematic exploration of human noncoding genetic variation at both cell type and single-nucleotide resolutions. We applied the framework to generate a comprehensive landscape of cell type-specific genetic variation across human tissues, and assessed how the inferred genetic regulation contributes globally to complex traits and diseases. We then extended the utility of the generated landscape to prioritize phenotype-driver cell types and to provide functional insights into genetic associations with diseases. To our knowledge, this is the first approach to achieve systematic noncoding variant interpretation at cell type levels, which may substantially promote the understanding of biology underlying phenotype-causal genetic variation. The data generated in this study represents an important resource. We developed the website (http://bis.zju.edu.cn/huatuo/, Supplementary Fig. 11) to facilitate usage of our data for the wide research community.

Evaluation of variant effects on specific genes with machine learning is complicated by the long-range interactions between promoter proximal regions and the distal regulatory elements. Computational methods based solely on the ab initio predictions from sequences may lead to an underestimation of regulatory effects for distal elements like enhancers. Instead, Huatuo gains insights into the genes perturbed by genetic variation through integrating standard eQTLs and cell cluster-ieQTLs, which allows us to pay more attention to distant gene regulation. Moreover, the genetic associations of (i)eQTLs are derived from practical measured data. Thus, they can provide independent statistical support for the ab initio sequence-based predictions. Altogether, our proposed framework overcomes the limitations arising from current technology and enables great potential for uncovering novel genetic regulation of gene expression with a high resolution.

Although our work refines the understanding of cell type-specific genetic regulation, it has limitations. Firstly, the validation of cell type-level genetic functional inference is currently challenging. With the rapid development of genome-editing technology, we look forward to future large-scale experimental datasets comparing with our computational results with functional cis-element analyses. Secondly, the relationships between de novo predicted functional variants and perturbed genes were inferred based on the LD statistics, which may not correspond to a direct regulatory effect. Thirdly, while the putative functional variants are predicted to have a cell type-specific impact on the transcription of surrounding genes, direct evidence showing the presence of associated (i)eQTL SNP-gene regulatory links in the same cell type is still lacking. Nevertheless, our framework offers an alternative way to assess the effects of noncoding variants in cell types/states of interest from readily accessible data. In the future, Huatuo could be applied to identify contribution of noncoding variants to diseases, uncover novel disease-causal genes and shed light on the cellular process critical for disease progression. We expect that Huatuo will provide valuable insights required for precision medicine and personalized healthcare.

## Methods

### Huatuo analytical workflow

The Huatuo framework consists of four main stages (Fig. 1). In the first stage, Huatuo employs a publicly available convolutional neural network (CNN) model, the 2019 version of DeepSEA^8^ (nicknamed Beluga, https://humanbase.readthedocs.io/en/latest/beluga.html) to integrate the sequence information from a wide sequence context. For ±20 kb genome regions centered on transcription start sites (TSSs) of 24,339 protein-coding, rRNA and lincRNA genes (Supplementary Data 1), Huatuo makes predictions for the collected chromatin factor features (2002 histone marks, TF-binding and DNase hypersensitivity profiles) of each 200-bp sequence bin. This deep-learning-based prediction generates a total of 400,400 features around the TSS of each gene (2002 chromatin feature*200 sequence bins).

In the second stage, by using scRNA-seq datasets, Huatuo builds cell cluster predictive models that capture the specific chromatin configuration within a cellular context. Specifically, the MAGIC algorithm (v.2.0.3)^29^ is used for imputation of the normalized single-cell expression matrix, with the recommended settings from its GitHub repository, to fill in missing genes and improve expressed gene numbers. The scRNA-seq data of multiple cells in the same cell cluster are next aggregated to generate the cell type-specific transcriptional expression profile. For the 24,339 genes of which the genomic sequence information around TSSs has been extracted by DeepSEA (Beluga), Huatuo applies the XGBoost algorithm^13^ to predict the log-transformed gene expression by the CNN outputs of chromatin features. When training the XGBoost model, the 990 genes located on chromosome 8 are assigned to the test set and the rest are assigned to the training set. Linear booster is applied by setting the parameter *booster* to *gblinear*. The maximum number of iterations is limited to 40 and early stop of the iteration process is tuned on to avoid overfitting (*early_stopping_rounds* = *10*). The step size shrinkage used for updating the model is set to 0.5 (*era* = 0.5). Linear regression is selected for the loss function (*objective = reg:linear*). The l2 regularization term on weights is increased by setting *lambda* to 100. The remaining hyperparameters are maintained as the default. Of note, the number of features used for gene expression predictions is enormously large (400,400 for each gene), whereas the sample size is relatively limited (24,339 genes). The reason behind the selection of XGBoost is that the regression models can suffer from the potential problems of multicollinearity and over-fitting by using shrinkage and regularization methods as a remedy. In addition, XGBoost offers the option to parallel the training process in an implicit style on a single machine^13^, which allows for training a set of cell cluster predictive models faster.

In the third stage, Huatuo estimates the effects of in silico mutagenesis at the level of chromatin by comparing the CNN predictions transformed by the fitted cell cluster models. Specifically, the differences in the CNN outputs of chromatin features between the reference and alternative allele sequences are first calculated to estimate the bulk-level variant effects. Next, the estimates are fed into the trained cell cluster models to predict the variant effects within specific cellular contexts. For an interested cell type, one-tailed t-tests are further performed to test whether the absolute values of variant effects predicted from the models of corresponding cell clusters are higher than those from other models. Based on the de novo predictions, Huatuo infers the nucleotide substitutions showing significantly higher effect for one major cell type as putative cell type-specific functional regulatory variants.

However, the XGBoost models may not be sufficient to investigate the roles of variants in regulating expression levels of specific genes, especially for the long-range interaction, due to that regulatory elements typically act in coordination to affect gene transcription^30^. Also, the results of de novo predictions are lack of further genetic evidence supporting. In the final stage, Huatuo performs an integration analysis that includes four steps. Firstly, the results of standard eQTLs and cell cluster-ieQTLs are obtained, independently from the variant prediction. Standard eQTLs in major human tissues can be downloaded from Genotype-Tissue Expression (GTEx) v8 data release (https://www.gtexportal.org/home/datasets). Cell cluster-ieQTL can be mapped using the provided scRNA-seq profiles and the genotype and transcriptomic datasets from GTEx. The detailed method is exemplified in the following subsection “Application of Huatuo to the Human Cell Landscape (HCL)”. Secondly, LD correlations are estimated with the exact same genotype matrix used for (i)eQTL mapping. Thirdly, variants in high linkage disequilibrium (LD, r2 > 0.8) with a candidate top eQTL or top ieQTL are merged using PLINK^31^ in each cis-window. If the putative functional variants from a cell cluster model can be merged with top (i)eQTLs from the matched tissues, they are considered to be supported by the association tests and causally linked to the (i)eQTL gene expression levels. Standard eQTLs and top ieQTLs mapped in the same tissue type are pooled together for the integration to provide a list of candidate genes whose transcription is likely to be disrupted by the regulatory variants. Finally, Huatuo outputs the putative cell type-specific functional variant mapped to at least one top (i)eQTL and the candidate genes cis-regulated by them.

### Application of Huatuo to the Human Cell Landscape (HCL)

In this work, we applied the proposed Huatuo framework to the single-cell transcriptome profiles of 357 cell clusters (a total of 44 major cell types according to the cell annotations) across 20 adult human tissues from the Human Cell Landscape (HCL)^7^. According to the Huatuo pipeline, we built a total of 357 cell cluster gene expression models (Supplementary Fig. 2). The model performance was measured by Pearson’s correlation coefficient (PCC) of gene expression levels computed across the 990 test set genes, which were excluded from the training set of both DeepSEA (Beluga) and XGBoost models. Across 357 cell clusters, the models achieved a 0.763 median PCC between the predicted and actually observed gene expression levels (Supplementary Fig. 2a, b). Some cell clusters of the kidneys, stomachs and transverse colons even obtained a PCC higher than 0.80. In addition, to assess the ability of models to predict cell type-specific gene expression, we compared the log fold change of predicted and observed expression levels in the focal cell cluster against all cell clusters and calculated the PCCs across the test set genes. The predictions of gene expression were found to recapitulate cell lineage specificity, showing more expression correspondence with within the same lineage and less correspondence between different lineages (Supplementary Fig. 2c). For the variants with an absolute value of predicted effect greater than 0.5, the top 2000 with the most differential prediction in each major HCL cell type were inferred as the cell type-specific functional variants.

To provide insight into SNP-gene interactions, ieQTL analyses were performed for each HCL cell cluster in the anatomically matched GTEx tissues (Supplementary Fig. 3 and Supplementary Data 2). First, the gene expression data from multiple cells in the same cell cluster were aggregated to make pseudo-cells, and then fed into CIBERSORTx^32^ (http://cibersortx.stanford.edu) to create a signature matrix file for the anatomically matched GTEx tissues. Next, the TPM gene expression data of GTEx tissues was used as the mixture file to be deconvoluted to estimate cellular compositions in an absolute mode. The CIBERSORTx S-mode (single cell mode) was applied for batch effect correction. Since the absolute score can be used for comparison across cell types^32^, we retained the cell clusters of which the estimated absolute proportions in more than a fifth of the samples were higher than 1 for downstream analyses. The estimated cellular fraction was inversely normally transformed to avoid influence of outliers. Then, we generated TMM-normalized gene expression in BED format for GTEx tissues. Specifically, similar to the regular eQTL discovery pipeline provided by the GTEx Consortium, gene-level expression data were TMM normalized and inversely normally transformed across samples. Genes were retained only if they satisfied the following two requirements: TPM ≥ 0.1 in ≥20% of samples and reads (unnormalized) ≥ 6 in ≥20% of samples. We combined PEER^33^ factors (Supplementary Data 2), the top 5 genetic principal components and other collected information (including ages, sexes) to represent the covariates in GTEx tissues. Finally, we identified ieQTLs for each cell cluster by testing the interaction term between SNP genotype and cellular fraction in the matched GTEx tissues through the following linear model:

$${Normalized}\,{Expression}\sim \beta 1\times {SNP}\,{dosage}:{Cellular}\,{fraction}+\beta 2\,\\ \times {SNP}\,{dosage}+\beta 3\times {Cellular}\,{fraction}+{Combined}\,{covariates}$$

The SNPs with a minor allele frequency (MAF) ≥ 0.05 in the samples and located on ±1 Mb genome regions centered on TSSs of each remained gene were used for the calculation, which was implemented in tensorQTL^34^. Nominal *p*-values of the interaction term were first Bonferroni adjusted for the effective number of independent variants in a cis-window, generating the eigenMT-adjusted *p*-value (pval_emt) for each top ieQTL^35^. The gene-level statistical significances were further Benjamini-Hochberg (BH) adjusted for all tested genes to assess the FDR (pval_adj_bh) at the genome-wide level.

Standard eQTLs were downloaded from the GTEx v8 data release (https://www.gtexportal.org/home/datasets). According to the original article^6^, the significance of each top standard eQTL (gene-level significance) was determined from empirical *p*-values, extrapolated from a Beta distribution fitted to adaptive permutations^36^. Similar to the ieQTL multiple testing correction, we then corrected the downloaded gene-level significance using the BH method to estimate genome-wide FDR.

Of note, we adopted a relaxed threshold of FDR < 0.4 to identify candidate top standard eQTLs and ieQTLs for integration analysis in the final stage of Huatuo. This cutoff for significance is due to the following points. Firstly, it is conservative to use Benjamini-Hochberg (BH) correction to control multiple tests at the genome-wide level. The reason is that thousands of features in a genome are tested against the null hypothesis in our association analysis. The BH procedure assumes π0 (i.e., the proportion of hypotheses for which the null is true) to be 1 in the set of association tests. However, a substantial fraction of genes is expected to be affected by genetic variants. And the association tests may actually originate from a mixture of both the null and the alternative hypothesis. Secondly, the Huatuo method integrates the results of de novo predictions and association tests. Only if one candidate top (i)eQTL (FDR < 0.4) are also located in high linkage disequilibrium (r2 > 0.8) with a variant showing putative transcriptional-regulation-disrupting effects from de novo prediction (predicted absolute value > 0.5), it is eventually inferred to be a variant-gene pair associated with the (i)eGene. Therefore, we believe that the integration analysis itself can effectively prevent false positives. Applying the traditional FDR cutoff of 0.05 to the pipeline may be too strict and lead to missed findings.

To further prove this, we present a benchmark example. We performed enrichment analyses for the top standard eQTLs and ieQTLs in arteries under four conditions: (a) FDR < 0.05 and predicted absolute value > 0.5; (b) FDR < 0.4 and predicted absolute value > 0.5; (c) FDR < 0.05; (d) FDR < 0.4 using chromatin state data. We downloaded dataset E065 of the matched tissue from the Roadmap Epigenome Project^37^ and performed ChromHMM^38^ to integrate different histone markers into chromatin states. Using a two-sided Fisher’s exact, we calculated the relative enrichment of (i)eQTLs under different conditions in cis-regulatory elements (cCREs, including promoters and enhancers) against the control groups. The control sets were constructed by randomly selecting the variants with matched chromosomes, MAFs and distances to TSS. When considering the results of association analysis only, we found the (i)eQTL group with an FDR < 0.05 was more enriched in cCREs compared to that with an FDR < 0.4, as expected. However, the (i)eQTL group jointly identified by FDR < 0.4 and predicted absolute value > 0.5 showed an even stronger enrichment than the group with an FDR < 0.05 alone (Supplementary Fig. 4). Overall, compared to a stringent FDR cutoff, our selected threshold of FDR < 0.4 is more suitable for use with Huatuo, as it strikes a sensible balance between the number of true and false positives.

### Comparisons between ab initio variant predictions and standard eQTLs

We examined whether the ab initio predictions of variant effects could reproduce the results of standard eQTLs that were mapped in GTEx bulk tissues. For one tested tissue, only the highest absolute value of predicted effect per variant from all cell cluster models were used for comparison. We compared the highest absolute values of variant predictions and the maximum z-scores (effect size/standard error) of eQTLs within the same LD block (r2  >  0.6) to calculate the correlation across all genes.

We then tested whether the ab initio variant predictions were able to distinguish the reported fine-mapped variants from the linked negative variants. We collected the fine-mapping results of GTEx eQTLs from a previous study^14^. To construct the control sets matched to the fine-mapped variant, we randomly selected their linked variants (r2  >  0.6) from those with the SuSiE^28^ results of posterior inclusion probability (PIP) less than 0.01. The highest absolute values of predicted effect per variant from the cell cluster models of the tested tissue were used to train logistic regression classifiers to determine whether a variant was the fine-mapped SNP of eQTLs.

### Identifications of known pathogenic variants

A set of noncoding variants regulating the expression levels of gene γ-globin (HBG) have been recorded as a cause of HPFH (Hereditary persistence of fetal hemoglobin) in Clinvar database^15^. We trained a cell type model using the expression profiles of HUDEP 2 cells^39^, and tested whether ab initio variant predictions were able to identify the recorded pathogenic variants. In silico mutagenesis (each reference base mutated to the other three bases) around the HBG TSS were computed one by one. Then, we constructed 10,000 null sets of variants matched to the pathogenic variants based on distance to the TSS. The fold difference was calculated as the mean absolute value of predicted effects for pathogenic variants divided by that for the null variants. The 95% confidence intervals were assessed with 10,000-time bootstrapping of the constructed null sets with replacement.

### Colocalization analysis

We downloaded 114 publicly available GWAS summary data from consortia and the UK Biobank^40^ (Supplementary Data 3). To determine whether the GWAS associations and cell cluster-ieQTLs or standard eQTLs share a causal variant, we used coloc^17^, a Bayesian framework, to perform colocalization analysis for all the cis-regulated genes, for which the nominal-*p*-values of top association were smaller than 1e−5. We examined the colocalization using the 1 Mb region centered on TSSs with the default prior probability in coloc and considered PP.H4 (representing the posterior probability of having the same causal variant between two association data) greater than 0.85 as an evidence of positive colocalization.

To validate the colocalization results, we downloaded a reported “silver standard” dataset including 1685 putative causal gene-trait pairs^18^, and tested whether they could be prioritized based on the PP.H4 values of colocalization. The dataset was curated based on the Online Mendelian Inheritance in Man (OMIM) database^41^, supposing that a more moderate alteration of Mendelian monogenic disease genes induced by regulatory variation may contribute to the risk of related polygenic GWAS traits^42^. The enrichment p-values of the curated gene-trait pairs in the colocalized signals were estimated based on the ranked PP.H4 values and 10,000 sample permutations implemented in the R package liger^43^.

### Comparisons between ab initio variant predictions and cell cluster-ieQTLs

To compare cell cluster-ieQTLs with ab initio variant predictions based on cell cluster models, we first used the smallest eigenMT-adjusted *p* value of top associations per gene (top ieQTLs) in each tissue to rank the cell cluster-ieQTLs. Then, we used the R package liger^43^ to estimate enrichment of the genetic loci linked to (r2 > 0.6) the predicted functional variants combined from cell cluster models of the tested tissue for the ranked list. The enrichment *p*-values were calculated as the probability of drawing an enrichment from the null sets through 10,000 permutations larger than that of the observed variant set.

Next, to obtain variants with cell type-specific regulatory effects based on the ab initio variant predictions, we trained additional tissue-level models using expression profiles of bulk tissues. The predicted functional variants were divided into two groups, depending on whether they had the absolute values of predicted effects from cell cluster models more than 1.96 standard deviation greater than those from the tissue-level models. We recalculated the enrichment of cell cluster-ieQTLs for the two group of variants, respectively. The normalized enrichment score (NES) was calculated as the enrichment of the observed variant set divided by the mean enrichment of 10,000 sample permutations.

### Benchmarking the genetic variation landscape against scATAC-seq data

To validate that the cell type-specific functional variants identified by Huatuo showed specific enrichment in the open chromatin regions of its corresponding cell type, we downloaded the chromatin accessibility profiles for 54 cell types from http://catlas.org/catlas_downloads/humantissues/, of which 42 matched cell types were used to perform enrichment analysis^19^. For each cell type, we calculated the overlap between the open chromatin regions estimated from the scATAC-seq data and the predicted functional variants. We then used the function AUCell_calcAUC from the R package AUCell^44^ to estimate the enrichment of the overlapped regions in the regions of differential chromatin accessibility for each cell type. This yielded a cell type by cell type table containing the area under the curve (AUC) scores representing the raw enrichment. The scores were then normalized row-wise and clustered with the Ward. D2 clustering algorithm.

### Examining global effects of identified variants on complex phenotypes

To verify that Huatuo enables identifications of the genetic regulation that serves as potential functional mechanisms underlying the GWAS associations of complex diseases and traits, we constructed logistic regression classifiers using the training dataset of the maximum absolute values of ab initio variant predictions and the strongest summary statistics of cell cluster-ieQTLs and standard eQTLs across all the investigated cell types. The observed variants with a GWAS *p*-values < 5e−8 were considered as positive association signals, and those with a *p*-value > 0.05 as negative signals. The classifiers were then used to distinguish between them for the GWASs with more than 20 defined positive association signals.

To investigate the global contributions of cell type-specific genetic regulation to complex diseases and traits, we used all the identified cell type-specific functional variants to prepare the annotation input files for LDSC v.1.0.1^20^ and estimated the SNP-based heritability enrichment for 114 different GWASs (https://github.com/bulik/ldsc/wiki). An additional category containing all SNPs were also added into the annotation following the instructions on the LDSC website. We used the genotype reference from the Phase 3 of the 1000 Genomes Project^45^ to estimate the LD scores. We corrected the enrichment p-values using the Bonferroni adjustment for the tested 114 GWASs. We also applied quantile-quantile (QQ) plot to examine the inflation of identified variants in the summary statistics of several GWASs below the Bonferroni-corrected significance threshold, implemented using the R package qqman v.0.1^46^.

### Cell type heritability enrichment

To interrogate the disease-relevant and trait-relevant cell types, we performed cell type-specific stratified LD score regression^21^ (https://github.com/bulik/ldsc/wiki/Cell type-specific-analyses) to estimate the marginal increases in the SNP-based heritability enrichment for each cell type. We jointly fit the following annotations: (1) the identified cell type-specific functional variants; (2) all of our predicted variants; (3) the baseline model downloaded from the LDSC website including 52 functional categories that are not specific to any cell type^20^. The analyses were performed to test whether the identified variants for a cell type showed significant enrichment for the per-SNP heritability, controlling for the functional categories of baseline model and the set of all predicted variants. The reported coefficient *p*-values were calculated from a one-sided test examining whether per-SNP heritability for the cell type annotation conditional on all other annotations would be positive. Genotype reference from the Phase 3 of the 1000 Genomes Project^45^ were used for LD score estimates. The enrichment score in Fig. 4b was defined as the signed log10-transformed values of heritability enrichment (proportion of SNP heritability / proportion of SNPs) multiplied by *p*-values.

### Huatuo-based variant-to-function mapping

To provide insights into the biological mechanisms of phenotype-causal genetic variation, we performed systematic mapping of causal variants identified by GWASs to the putative cell type-specific genetic regulation from Huatuo at single-nucleotide resolutions. For each of the 100 kb GWAS-associated genome regions (*p*-value < 1e−5) that contain an identified regulatory variant, we performed fine mapping analysis with SuSiE^28^ (https://stephenslab.github.io/susieR/) based on the summary statistics of GWASs to acquire the 95% credible sets of fine-mapped SNPs. The 95% credible sets were considered as the smallest variant set to have a causal variant driving the observed GWAS association signals. The correlation matrix in genetics (LD matrix) were generated using --r square in PLINK v.1.9^31^. We considered the cell type-specific genetic regulation as the potential intermediate phenotypes that cause the phenotype variances, when the Huatuo identified regulatory variants were in the estimated 95% credible sets of disease-causal and trait-causal SNPs. The circos plot in Fig. 4e was drawn using the OmicStudio tools at https://www.omicstudio.cn/tool/.

### Reporting summary

Further information on research design is available in the Nature Portfolio Reporting Summary linked to this article.

## Supplementary information

Supplementary Information

Description of Additional Supplementary Files

Supplementary Data 1

Supplementary Data 2

Supplementary Data 3

Supplementary Data 4

Supplementary Data 5

Supplementary Data 6

Supplementary Data 7

Supplementary Data 8

Reporting Summary

## Data Availability

All datasets analyzed in this study were published previously. All GTEx open-access data are available on the GTEx Portal (https://gtexportal.org/home/datasets). GTEx protected data are available via dbGaP (accession phs000424.v8). The Human Cell Landscape data are available at https://db.cngb.org/HCL/. Download links of all GWAS summary data are summarized in Supplementary Data 3. Single-cell chromatin accessibility profiles (scATAC-seq) used for validating cell-type specificity of identified variants are available at http://catlas.org/catlas_downloads/humantissues/. The pathogenic variants for HPFH can be downloaded from Clinvar database (https://www.ncbi.nlm.nih.gov/clinvar/). The datasets E065 from the Roadmap Epigenome Project are available at https://personal.broadinstitute.org/anshul/projects/roadmap/alignments/consolidated/.Data from the main figures are available in the Supplementary Information. Huatuo identified cell type-specific genetic variation of gene regulation and variant-to-function mapping of GWAS associations can be accessed at http://bis.zju.edu.cn/huatuo/.

## Code availability

All Python scripts and R scripts used for data analysis are available at GitHub (https://github.com/ggjlab/huatuo) and Zenodo (https://zenodo.org/record/7968802)^47^.
