# Supplementary material for: An analytical framework for decoding cell type-specific genetic variation of gene regulation
Source: Nat Commun. 2023 Jun 30;14:3884. doi: 10.1038/s41467-023-39538-7 (PMC10313894; doi:10.1038/s41467-023-39538-7)
Supplement: Supplementary file 1 — Supplementary Information [file 41467_2023_39538_MOESM1_ESM.pdf]

Supplementary Information

An analytical framework for decoding cell type-specific genetic variation of gene regulation

Yanyu Xiao[1,2†], Jingjing Wang[1,2†*], Jiaqi Li[1†], Peijing Zhang[1,2†], Jingyu Li[1], Yincong Zhou[3], Qing Zhou[4], Ming Chen[3], Xin Sheng[2], Zhihong Liu[2], Xiaoping Han[1,5*], Guoji Guo[1,2,5,6*]

[1]Center for Stem Cell and Regenerative Medicine, and Bone Marrow Transplantation Center of the First Affiliated Hospital, Zhejiang University School of Medicine, Hangzhou, Zhejiang 310000, China.

[2]Liangzhu Laboratory, Zhejiang University Medical Center, Hangzhou, Zhejiang 311121, China.

[3]College of Life Sciences, Zhejiang University, Hangzhou, Zhejiang 310003, China.

[4]Life Sciences Institute, Zhejiang University, Hang Zhou, Zhejiang 310058, China.

[5]Zhejiang Provincial Key Lab for Tissue Engineering and Regenerative Medicine, Dr. Li Dak Sum & Yip Yio Chin Center for Stem Cell and Regenerative Medicine, Hangzhou, Zhejiang 310058, China.

[6]Zhejiang University-University of Edinburgh Institute, Zhejiang University School of Medicine, Zhejiang University, Hangzhou 314400, China.

[†]These authors contributed equally to this work.

[*]Correspondence author. Email: ggj@zju.edu.cn (G.G.); xhan@zju.edu.cn (X.H.); jingjingw@zju.edu.cn (J.W.)

This PDF includes:

Supplementary Tables 1 and Supplementary Figures 1-11

Supplementary Table

Supplementary Table 1. Replication summary statistics for the OneK1K dataset.

ieQTL cell type	OneK1K cell type	Tested ieQTL number	Replication (%)	Replication of other ieQTL cell types (%)	Replication across OneK1K cell types (%)
T cell	CD8 NC, CD8 ET, CD8 S100B, CD4 NC, CD4 ET, CD4 SOX4	83	55.4	28.1	62.7
B cell	B IN, B Mem, Plasma	50	26	14.8	40
NK cell	NK R, NK	24	50	21	62.5
Monocyte	Mono NC, Mono C	313	16.6	15.3	38.7

Supplementary Figures

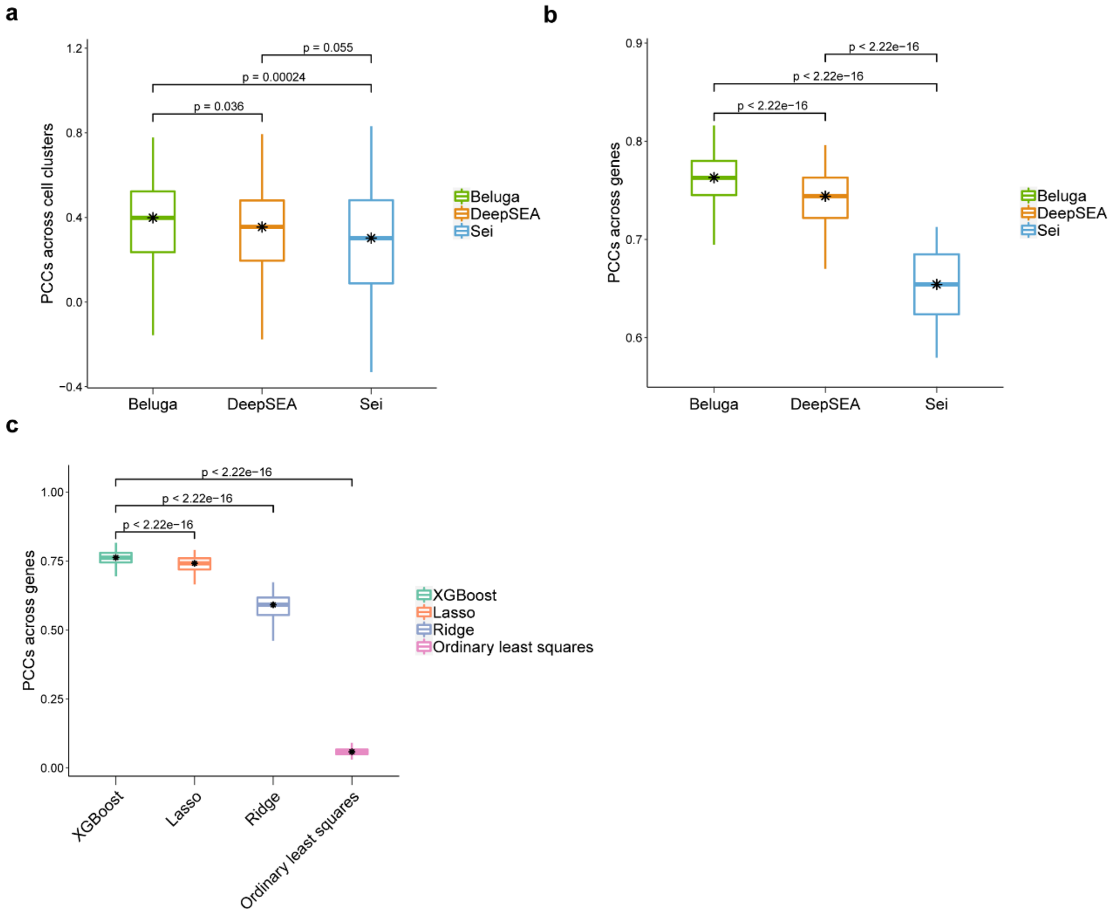

Supplementary Fig. 1. Benchmarks testing downstream ability to predict gene expression. To select a more suitable sequence model for Huatuo, we used the chromatin features generated from DeepSEA[1], DeepSEA Beluga[2] and Sei[3] to train predictive models for 357 HCL cell clusters, respectively. Specifically, we employed the CNNs to integrate the sequence information for ±20 kb genome regions centered on transcription start sites (TSSs). For the three CNN models, 919, 2,002 and 21,907 epigenomic features were predicted for each 200-bp genomic region using sequence window sizes of 1,000bp, 2,000bp and 4,096bp as inputs, respectively. The prediction yielded a total of 183,800, 400,400 and 4,381,400 features for each gene, which were further used to build XGBoost model for each cell cluster (n=357 for each group). The model performance (n=357 for each group) was measured by Pearson’s correlations (PCCs) of gene expression computed across genes for each cell cluster (**a**) or across

cell clusters for each test gene (**b**). To select a more suitable regression model for Huatuo, we trained XGBoost[4], lasso, ridge and ordinary least-squares linear regression to predict gene expression from the chromatin features generated by DeepSEA Beluga, respectively. The test set performance of cell cluster models was measured by Pearson's correlations of gene expression computed across genes for each cell cluster (c). Box plots depict the interquartile range (IQR), whiskers depict 1.5× IQR. Statistical test: two-sided Wilcoxon rank sum test.

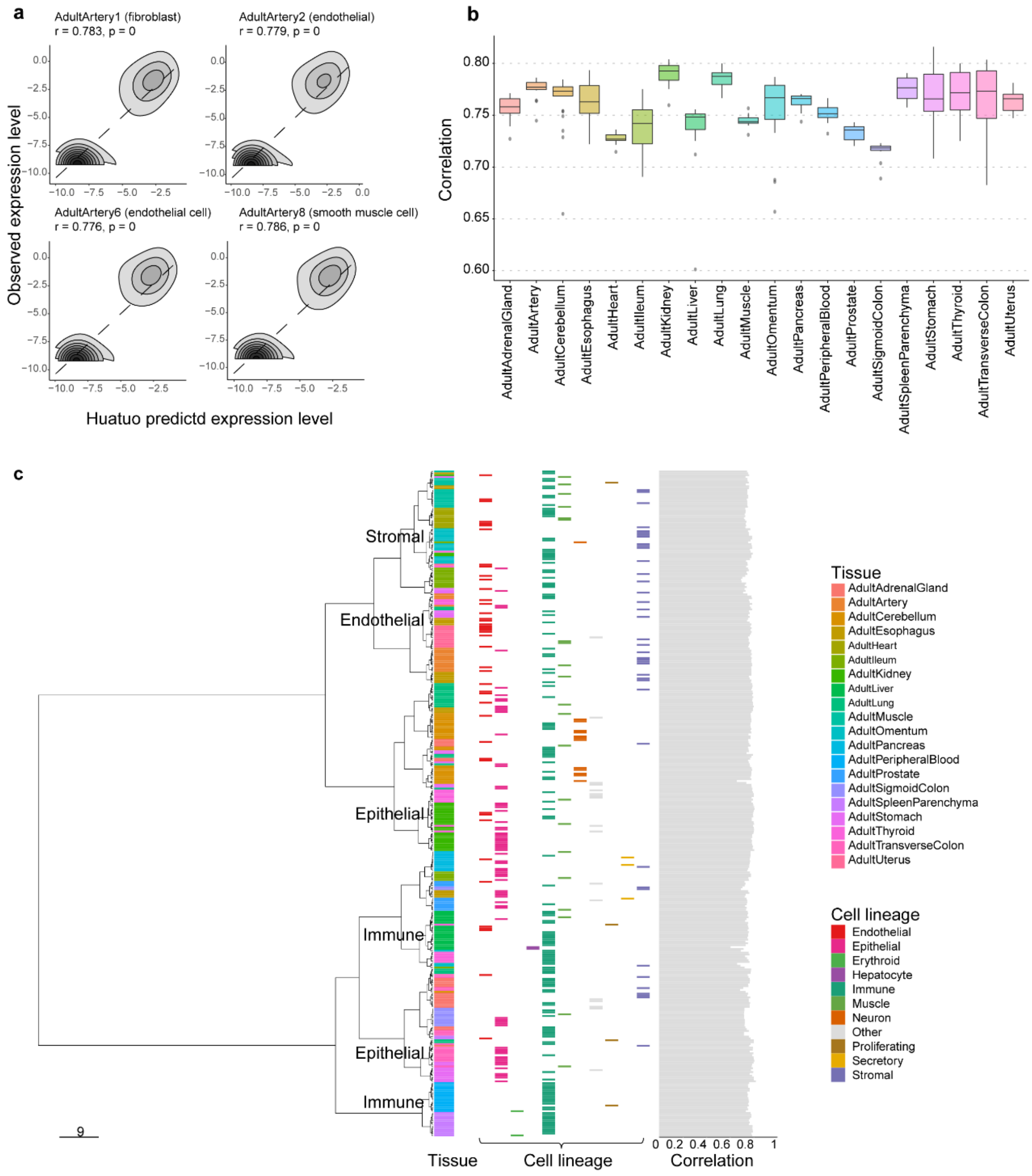

Supplementary Fig. 2. Gene expression predictions across the Human Cell Landscape (HCL) based on Huatuo cell cluster models. (**a**) Plots for predicted versus actually observed log gene expression levels in arterial cell clusters showing good performances of the prediction models for single-cell mRNA-seq data. The p-value was obtained from the two-sided Pearson correlation test. (**b**) Boxplots showing the overall predictive performances among 357 cell clusters across 20 tissues. Pearson correlations between predicted and observed gene expression are displayed. Box plots depict the

IQR, whiskers depict 1.5× IQR. (**c**) Dendrogram describing the relationship of 357 cell clusters according to the pairwise Pearson correlations between predicted and observed cell type-specific expression profiles, which are measured by log fold change of the gene expression levels in the focal cell cluster over the average levels across 357 cell clusters using predicted and observed values, respectively. The tissue of each cell cluster is indicated above the tree as colored rows. The middle panel indicates the cell lineage for each cell cluster. The right panel indicates the correlation between predicted and observed cell type-specific expression profiles for each cell cluster. Main branches, corresponding to the taxonomy, are annotated with cell lineage.

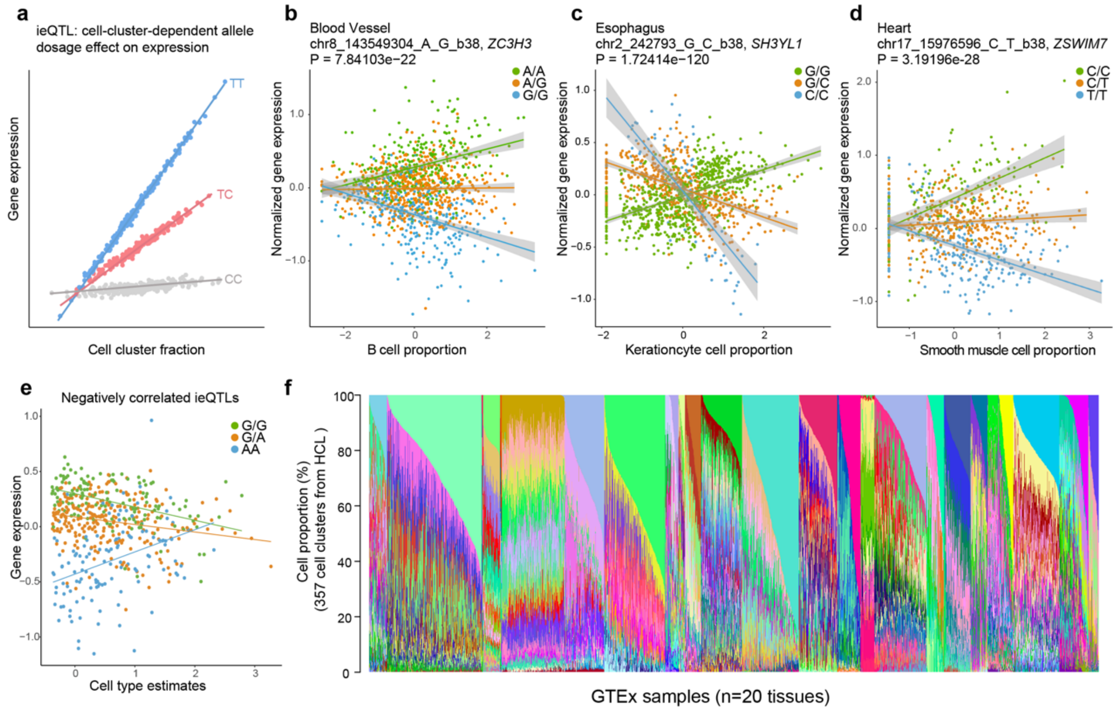

Supplementary Fig. 3. Mapping cell cluster-ieQTLs using GTEx genotypes and transcriptomic data. (**a**) The scheme of cell cluster-ieQTLs. (**b-d**) Examples for cell cluster-ieQTLs in several adult tissues (arteries, esophagus and hearts) showing how regulatory effects of noncoding variants in GTEx tissues are modified by the estimated proportions of HCL cell clusters based on *in silico* deconvolution. The x-axis and y-axis represent the normalized estimated cell cluster proportion and gene expression levels in the bulk tissues, respectively. Each dot refers to an individual in the GTEx cohort, and dot colors indicate the genotype at this locus. Colored lines represent means and the shadings represent SEMs. The two-sided p-value was generated for SNP-gene pairs using tensorQTL[5] with the cell cluster-ieQTL linear regression model. (**e**) Examples for negatively correlated cell cluster-ieQTLs, for which the genotype main effects in the samples with cell cluster estimates below the 25th percentile are stronger than those in the tissue samples with cell cluster estimates above 75th percentile. (**f**) Relative proportion estimates for 357 HCL cell clusters across GTEx tissue samples.

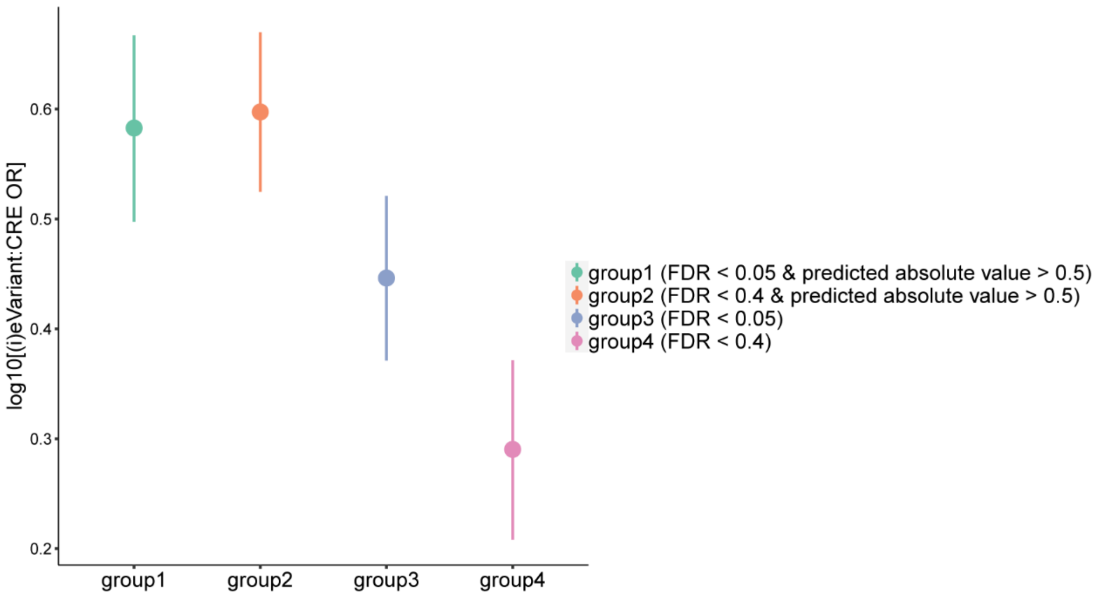

Supplementary Fig. 4. Benchmarking Huatuo results for given different (i)eQTL FDR thresholds against ChIP-seq data. Enrichment of eVariants (y-axis) in tissue-matched cis-regulatory elements (cCREs) for four group of identified top standard eQTLs and ieQTLs discovered for each gene (x-axis). Bars indicate 95% CI.

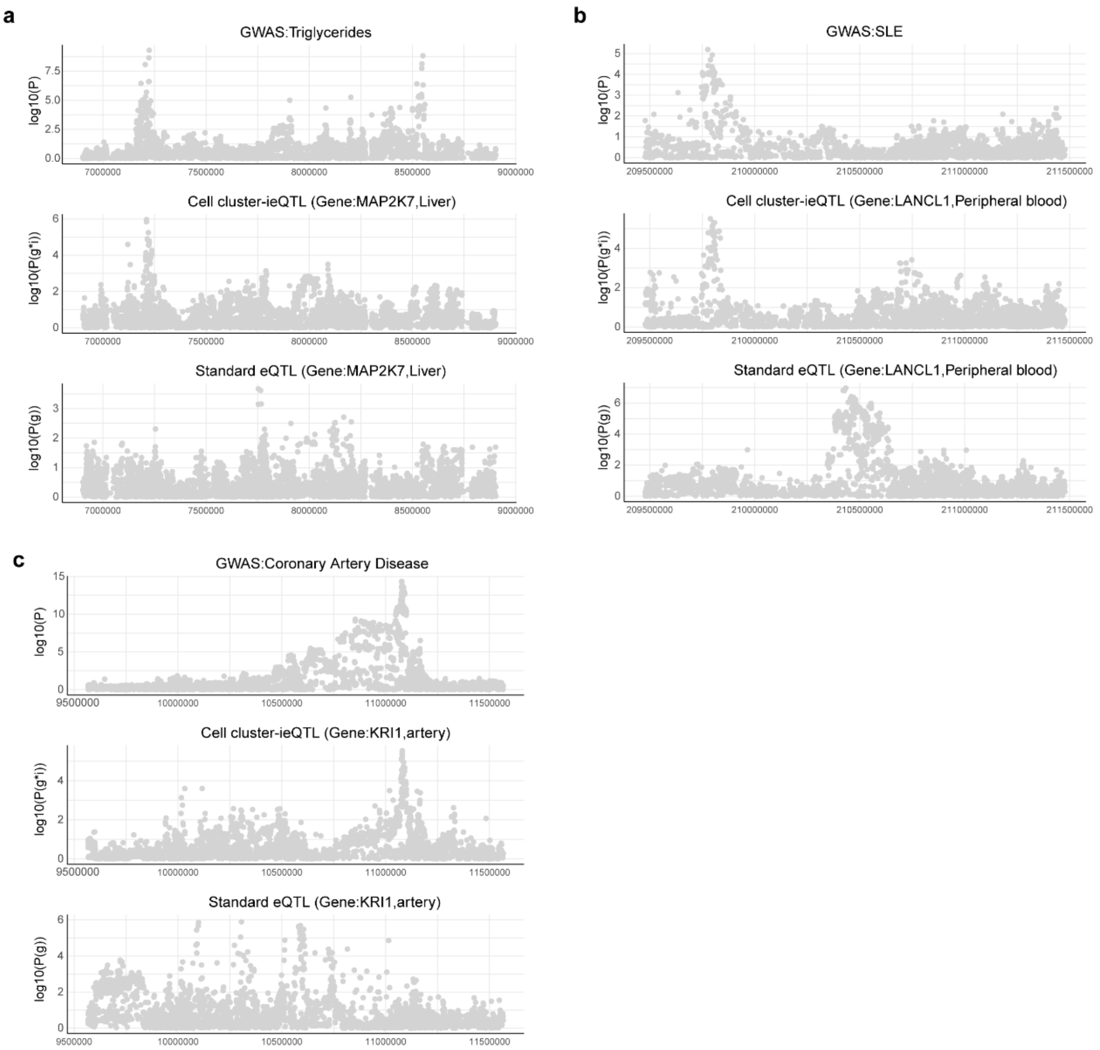

Supplementary Fig. 5. Cell cluster-ieQTLs allows discovery of novel regulatory loci associated with complex traits and diseases. Several examples of GWAS signals for (a) triglyceride, (b) systemic lupus erythematosus, and (c) coronary artery disease that can only be colocalized with cell cluster-ieQTLs. Top panels: Manhattan plots of complex phenotype. The two-sided p-value was obtained from the GWAS study; middle panels: Manhattan plots of cell cluster-ieQTLs. The two-sided p-value was generated for SNP-gene pairs using tensorQTL with the cell cluster-ieQTL linear regression model; bottom panels: The two-sided p-value was downloaded from the GTEx portal (Data Source: GTEx Analysis Release V8)

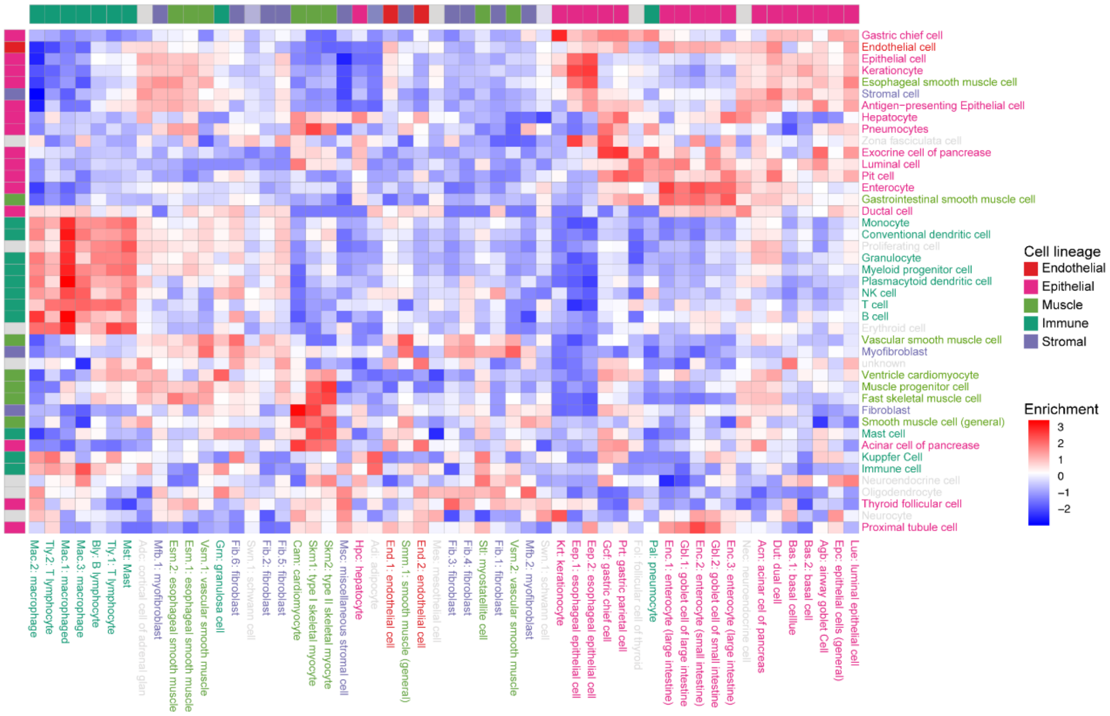

Supplementary Fig. 6. Cell cluster-ieQTL SNPs were enriched in the regulatory regions of cell types with poorer cell type specificity. The heatmap shows z-scores for the enrichment of cell cluster-ieQTL SNPs of each investigated HCL cell type (y-axis) in the differential chromatin accessibility of each cell type from an external scATAC-seq dataset (x-axis). The enrichment was evaluated using AUC scores similar to Fig. 3b, reflecting the extent of agreement between the cell cluster-ieQTL SNPs and the observed cell type-specific regulatory regions. The color-coding of labels on both the x-axis and y-axis indicates the cell lineage assignment of investigated cell types.

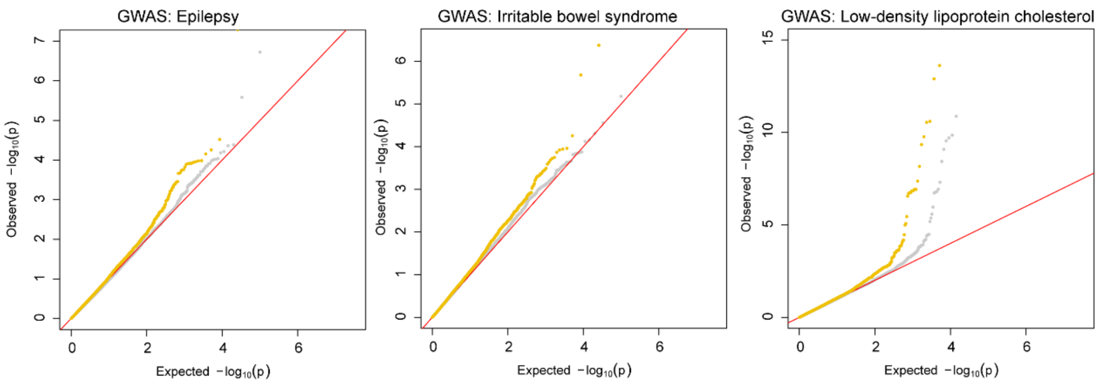

Supplementary Fig. 7. Cell type-specific genetic regulation contributes globally to complex traits and diseases. Several examples of GWAS traits that exhibit non-significant SNP-based heritability enrichment of Huatuo-inferred cell type-specific regulatory variants at Bonferroni levels. Quantile–quantile (Q–Q) plots showing inflations of Huatuo-inferred variants in GWAS summary statistics for epilepsy (left panel, Bonferroni-corrected LDSC enrichment $p = 0.67$), irritable bowel syndrome (middle panel, Bonferroni-corrected LDSC enrichment $p = 1$), and low-density lipoprotein cholesterol (right panel, Bonferroni-corrected LDSC enrichment $p = 0.35$). The two-sided p-value was obtained from the GWAS study.

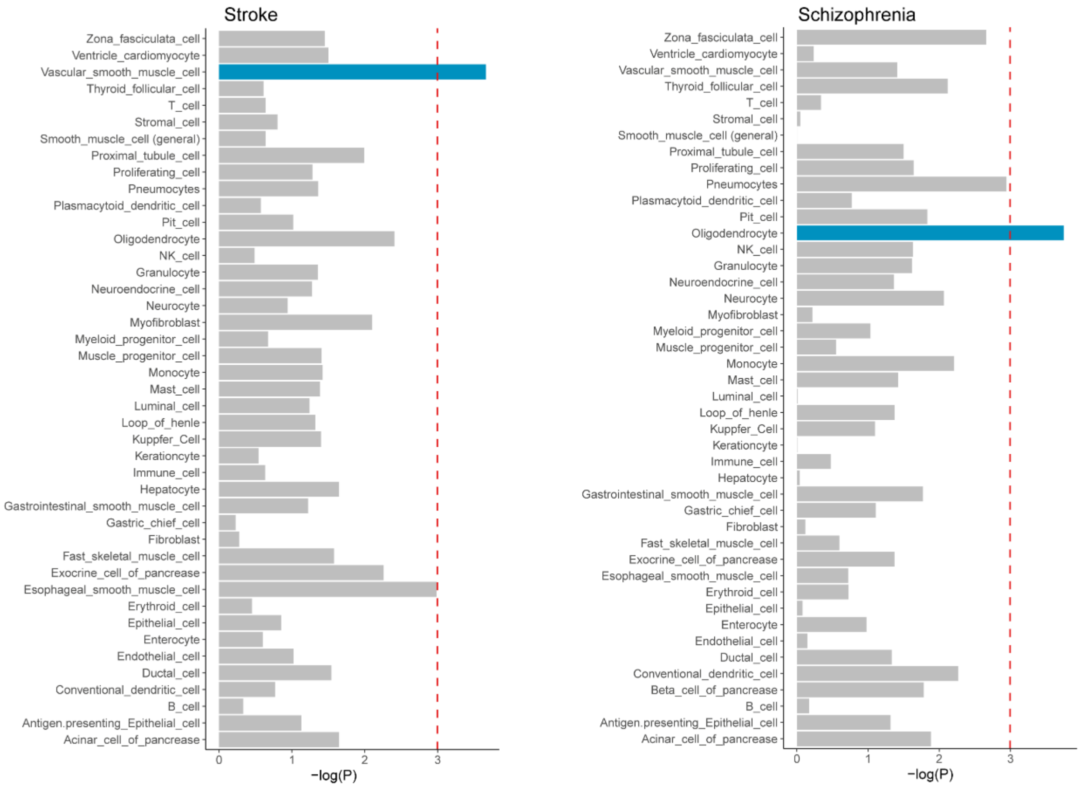

Supplementary Fig. 8. Huatuo-based prioritizations reveal well-known associations between complex phenotypes and cell types. Applying Huatuo to prioritize disease-relevant cell types in stroke (left panel) and schizophrenia (right panel). The y axis represents different cell types, and the x axis indicates the log10-transformed p-values of cell type heritability enrichment based on stratified LD score regression analysis. The top cell types (colored blue) are consistent with existing knowledge.

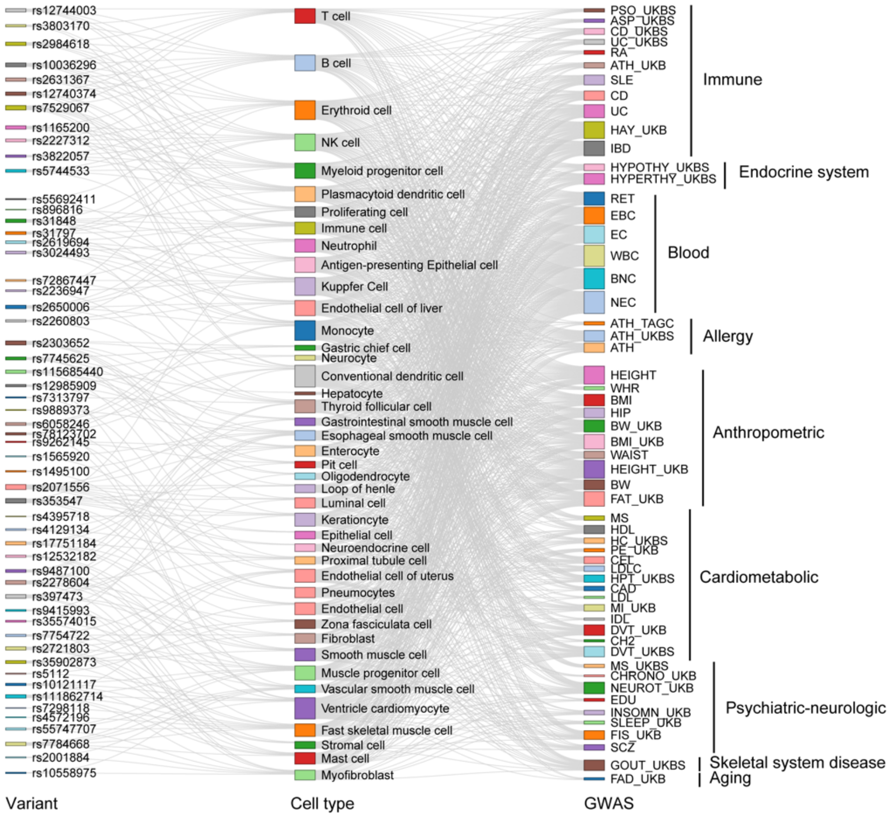

Supplementary Fig. 9. Huatuo uncovers cell type-specific genetic regulation contributing to genetic risk for a broad range of complex phenotypes. Sankey diagram displaying the connectivity between complex phenotypes (right), cell types (middle) and the putative functional regulatory variants (left) that are thought to underlie trait-causal and disease-causal genetic variation from at least 3 GWASs for each GWAS category. GWAS abbreviation: Supplementary Data 3.

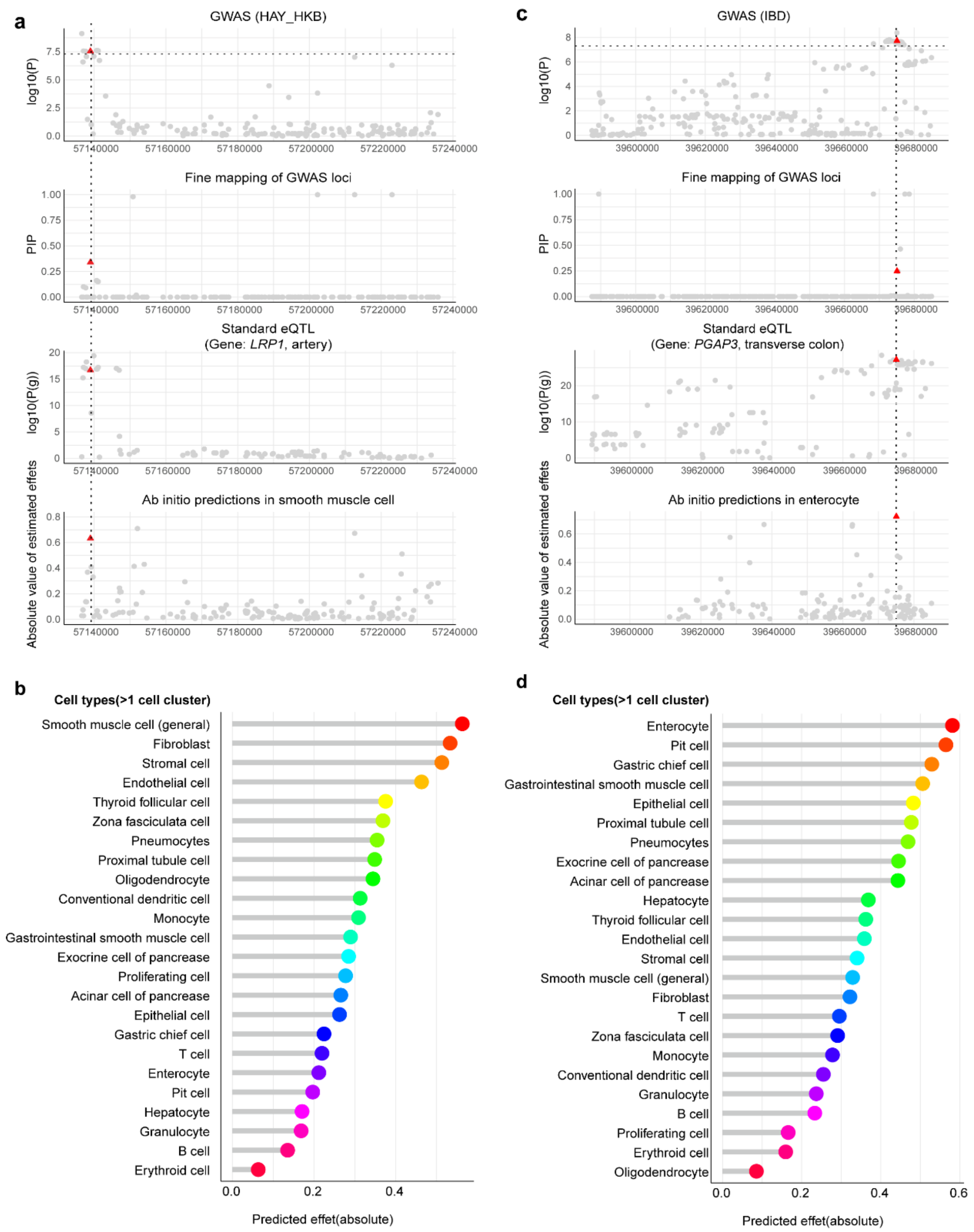

Supplementary Fig. 10. Examples of Huatuo-based variant-to-function mapping. Huatuo-based variant-to-function mapping for hayfever/allergic rhinitis (a, b) and inflammatory bowel disease (IMD) (c, d). The plots are arranged similarly to Fig. 4c and d.

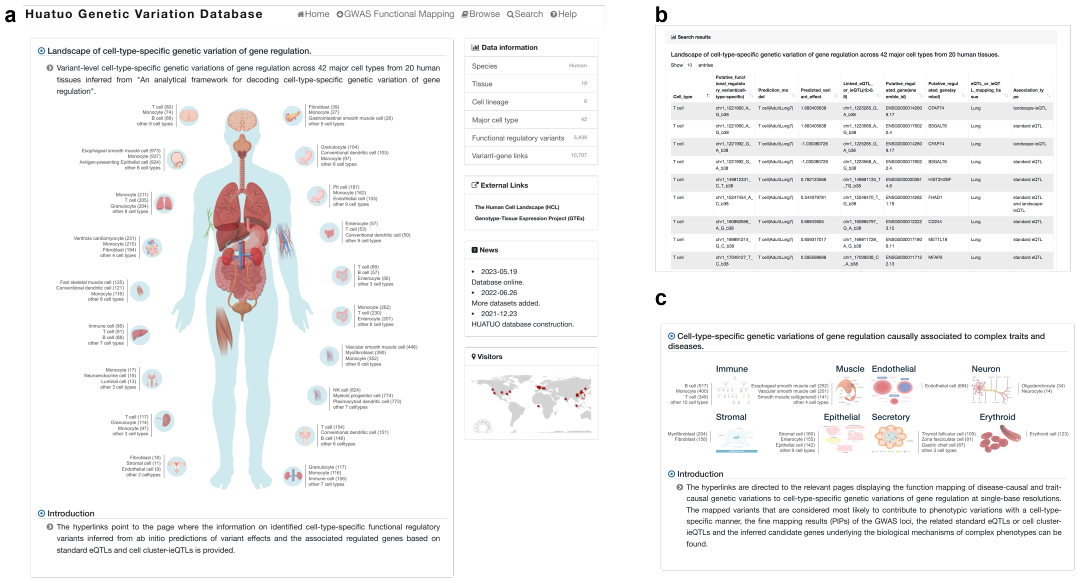

Supplementary Fig. 11. A screenshot of Huatuo Genetic Variation Database. The website of Huatuo (http://bis.zju.edu.cn/huatuo/) is developed for browsing the generated landscape of putative cell type-specific genetic regulation across 44 major cell types from 20 human tissues and complex traits and diseases associated with them (including the results of Bayesian colocalization and genetic regulation underlying phenotype-causal genetic variation). (a) The home page provides a global view of the human cell type-specific genetic variations of gene regulation inferred by Huatuo. The map in the panel (lower right) was generated using www.revolvermaps.com. (b) The page displays the search results corresponding to a specific cell type when the hyperlink from the home page is clicked. (c) The browse page presents the variant-level function mapping results of GWASs in a hierarchical classification of cell lineages and major cell types.

Supplementary References

1 Zhou, J. & Troyanskaya, O. G. Predicting effects of noncoding variants with deep learning-based sequence model. *Nat Methods* **12**, 931-934, doi:10.1038/nmeth.3547 (2015).

2 Zhou, J. *et al.* Deep learning sequence-based ab initio prediction of variant effects on expression and disease risk. *Nat Genet* **50**, 1171-1179, doi:10.1038/s41588-018-0160-6 (2018).

3 Chen, K. M., Wong, A. K., Troyanskaya, O. G. & Zhou, J. A sequence-based global map of regulatory activity for deciphering human genetics. *Nat Genet* **54**, 940-949, doi:10.1038/s41588-022-01102-2 (2022).

4 Chen, T. & Guestrin, C. XGBoost: A Scalable Tree Boosting System. *Proceedings of the 22nd ACM SIGKDD International Conference on Knowledge Discovery and Data Mining* (2016).

5 Taylor-Weiner, A. *et al.* Scaling computational genomics to millions of individuals with GPUs. *Genome Biology* **20**, 228, doi:10.1186/s13059-019-1836-7 (2019).
