# Supplementary material for: An analytical framework for decoding cell type-specific genetic variation of gene regulation
Source: Nat Commun. 2023 Jun 30;14:3884. doi: 10.1038/s41467-023-39538-7 (PMC10313894; doi:10.1038/s41467-023-39538-7)
Supplement: Supplementary file 2 — Description of Additional Supplementary Files [file 41467_2023_39538_MOESM2_ESM.pdf]

Description of Additional Supplementary Files

File name: Supplementary Data 1

Description: Genes used for building cell cluster models.

File name: Supplementary Data 2

Description: Information about ieQTL mapping and top associations per gene of the cell cluster-ieQTLs mapped for the HCL data across 20 adult human tissues. We reported the test results of all candidate variant-gene links with a genome-wide FDR less than 0.4.

File name: Supplementary Data 3

Description: The metadata and download links of 114 harmonized and imputed GWASs.

File name: Supplementary Data 4

Description: Colocalizations between 114 GWASs and cell cluster-ieQTLs (PP.H4 > 0.85).

File name: Supplementary Data 5

Description: Huatuo-inferred cell type-specific genetic regulation of 44 major cell types. For each cell type, we reported the putative cell type-specific functional regulatory variant, the *ab initio* predictions of variant effects based on corresponding cell cluster model, the linked standard eQTLs or landscape ieQTLs ($r2 > 0.8$, genome-wide FDR < 0.4) and the putative regulated genes.

File name: Supplementary Data 6

Description: The discrimination accuracy between positive and negative GWAS signals based on Huatuo-inferred variant effects. We reported the AUROC scores for the GWASs with more than 20 positive signals among the observed variants.

File name: Supplementary Data 7

Description: Heritability enrichment of Huatuo-inferred cell type-specific functional regulatory variants for 114 GWAS traits based on stratified LD score regression analysis.

File name: Supplementary Data 8

Description: The results of Huatuo-based variant-to-function mapping. In total, 699 cell type-specific functional regulatory variants were identified to contribute to genetic risk

for complex traits and diseases. 672 genes whose expression levels show correlation with them were also suggested to affect the biology relevant to the phenotypes. For each complex phenotype, we reported mapped functional regulatory variants, fine-mapping posterior inclusion probability (PIP) of the mapped variants at GWAS loci and standard eQTLs or landscape ieQTLs linked to the mapped variants (r2 > 0.8).
