# Supplementary material for: An analytical framework for decoding cell type-specific genetic variation of gene regulation
Source: Nat Commun. 2023 Jun 30;14:3884. doi: 10.1038/s41467-023-39538-7 (PMC10313894; doi:10.1038/s41467-023-39538-7)
Supplement: Supplementary file 11 — Reporting Summary [file 41467_2023_39538_MOESM11_ESM.pdf]

Corresponding author(s): Guoji Guo; Xiaoping Han; Jingjing Wang

Last updated by author(s): Jun 9, 2023

Reporting Summary

Nature Portfolio wishes to improve the reproducibility of the work that we publish. This form provides structure for consistency and transparency in reporting. For further information on Nature Portfolio policies, see our Editorial Policies and the Editorial Policy Checklist.

Statistics

For all statistical analyses, confirm that the following items are present in the figure legend, table legend, main text, or Methods section.

n/a	Confirmed	
☐	☒	The exact sample size (*n*) for each experimental group/condition, given as a discrete number and unit of measurement
☐	☒	A statement on whether measurements were taken from distinct samples or whether the same sample was measured repeatedly
☐	☒	The statistical test(s) used AND whether they are one- or two-sided<br>*Only common tests should be described solely by name; describe more complex techniques in the Methods section.*
☐	☒	A description of all covariates tested
☐	☒	A description of any assumptions or corrections, such as tests of normality and adjustment for multiple comparisons
☐	☒	A full description of the statistical parameters including central tendency (e.g. means) or other basic estimates (e.g. regression coefficient) AND variation (e.g. standard deviation) or associated estimates of uncertainty (e.g. confidence intervals)
☐	☒	For null hypothesis testing, the test statistic (e.g. *F*, *t*, *r*) with confidence intervals, effect sizes, degrees of freedom and *P* value noted<br>*Give P values as exact values whenever suitable.*
☐	☒	For Bayesian analysis, information on the choice of priors and Markov chain Monte Carlo settings
☐	☒	For hierarchical and complex designs, identification of the appropriate level for tests and full reporting of outcomes
☐	☒	Estimates of effect sizes (e.g. Cohen's *d*, Pearson's *r*), indicating how they were calculated

Our web collection on statistics for biologists contains articles on many of the points above.

Software and code

Policy information about availability of computer code

Data collection	Aspera (v3.7.4) was used as a tool to download GTEx protected data.
Data analysis	CIBERSORTx (http://cibersortx.stanford.edu) was used for in silico deconvolution; tensorQTL (v1.0.5) was used to calculate genetic interaction effect; R package PEER (v1.3) was used to correct for unobserved confounders in the expression data for QTL mapping; LDSC (v1.0.1) was used to partition heritability; R package SuSiE (v0.11.92) was used for statistic fine mapping; PLINK (v0.1.0) was used to process genotype data; R package AUCell (v1.5.2) was used for enrichment of peaks in chromatin accessibility; R package coloc (v5.1.0) was used for colocalization analysis; R package liger (v2.0.1) was used for enrichment estimation; ChromHMM (v1.23) was used to integrate different histone markers into chromatin states.<br>The source code for running the Huatuo framework is available at https://github.com/ggjlab/huatuo/.

For manuscripts utilizing custom algorithms or software that are central to the research but not yet described in published literature, software must be made available to editors and reviewers. We strongly encourage code deposition in a community repository (e.g. GitHub). See the Nature Portfolio guidelines for submitting code & software for further information.

Data

Policy information about availability of data

All manuscripts must include a data availability statement. This statement should provide the following information, where applicable:

- Accession codes, unique identifiers, or web links for publicly available datasets
- A description of any restrictions on data availability
- For clinical datasets or third party data, please ensure that the statement adheres to our policy

All datasets analyzed in this study were published previously. All GTEx open-access data are available on the GTEx Portal (https://gtexportal.org/home/datasets). GTEx protected data are available via dbGaP (accession phs000424.v8). The Human Cell Landscape data are available at https://db.cngb.org/HCL/. Download links of all GWAS summary data are summarized in Supplementary Data 3. Single-cell chromatin accessibility profiles (scATAC-seq) used for validating cell-type specificity of identified variants are available at http://catlas.org/catlas_downloads/humantissues/. The pathogenic variants for HPFH can be downloaded from Clinvar database (https://www.ncbi.nlm.nih.gov/clinvar/). The datasets E065 from the Roadmap Epigenome Project are available at https://personal.broadinstitute.org/anshul/projects/roadmap/alignments/consolidated/. Data from the main figures are available in the Supplementary Information. Huatuo identified cell type-specific genetic variation of gene regulation and variant-to-function mapping of GWAS associations can be accessed at http://bis.zju.edu.cn/huatuo/.

Human research participants

Policy information about studies involving human research participants and Sex and Gender in Research.

Reporting on sex and gender	No experiments in this study.
Population characteristics	No experiments in this study.
Recruitment	No experiments in this study.
Ethics oversight	No experiments in this study.

Note that full information on the approval of the study protocol must also be provided in the manuscript.

Field-specific reporting

Please select the one below that is the best fit for your research. If you are not sure, read the appropriate sections before making your selection.

☒ Life sciences ☐ Behavioural & social sciences ☐ Ecological, evolutionary & environmental sciences

For a reference copy of the document with all sections, see nature.com/documents/nr-reporting-summary-flat.pdf

Life sciences study design

All studies must disclose on these points even when the disclosure is negative.

Sample size	In our cell cluster-ieQTL analysis, we exclusively utilized GTEx tissues that had corresponding tissue types in the Human Cell Landscape data and also had reported standard eQTL results in the GTEx portal, which indicated that the sample size was sufficient. We performed the analysis on a total of 10,280 GTEx samples derived from 944 post-mortem donors, all of whom had both RNA-seq and genotype data available. No other specific statistical methods were used to predetermine the sample size.
Data exclusions	The cell clusters of Human Cell Landscape with the lowest quintile of estimated fraction in a matched GTEx tissue were excluded to compute landscape-ieQTLs.
Replication	Across the 357 cell cluster models used, we observed a strong agreement between the predicted gene expression levels and the observed scRNA-seq data, indicating the model's ability to make accurate quantitative predictions. Furthermore, the predicted variant effects (allelic impact) obtained from the cell cluster models consistently aligned with the eQTL regulatory effects reported by the GTEx Consortium across 20 different tissues. These findings also demonstrated agreement with the fine-mapped GTEx eQTLs for both distal and proximal regulatory regions, as well as with pathogenic mutations documented in the Clinvar database.<br><br>For most investigated cell types, the predicted regulatory variants displayed a higher enrichment in the open chromatin regions specific to the corresponding cell type. This observation suggests a consistent relationship between the identified cell type-specific regulatory variants and the corresponding regulatory regions specific to those cell types.<br><br>The identified cell cluster-ieQTLs showed a higher overlap with previously reported ieQTLs compared to standard eQTLs, which highlights the reproducibility of ieQTL studies. These cell cluster-ieQTLs also exhibited a greater enrichment in variants predicted to have higher effects based on the cell cluster models compared to tissue models. This finding further supports the agreement between variant predictions and the discovery of cell type-specific genetic regulation. In addition, the colocalization analysis between cell cluster-ieQTLs and GWASs revealed

colocalized signals that aligned with the "silver standard" from the OMIM database.

On average, 37% of the identified cell type-specific ieQTLs for B cells, T cells, monocytes, and NK cells were replicated in their corresponding matched OneK1K cell types.

Randomization

In our application of Huatuo to the Human Cell Landscape, we employed a random selection process to choose 500 cells from each cell cluster, as long as the cell cluster contained more than 500 cells. These selected cells were then aggregated to create pseudo-cells for further analysis.

In order to evaluate the ability of Huatuo to identify known pathogenic variants for HPFH (Hereditary Persistence of Fetal Hemoglobin), we compared the predicted variant effects of these known pathogenic variants to those of randomly selected variants. The randomly selected variants were chosen to have a matched distance to the transcription start site (TSS) around the HBG gene.

During the functional enrichment analysis for both standard eQTLs and ieQTLs under different conditions, we constructed control sets by randomly selecting variants that shared the same chromosome, minor allele frequencies (MAFs), and distances to the TSS as the variants being tested. This control set allowed us to compare the enrichment results and assess the significance of the identified eQTLs.

To assess whether the de novo variant predictions were capable of identifying fine-mapped eQTLs, we randomly selected negative variants that had a posterior inclusion probability (PIP) of less than 0.01 according to the SuSiE results, and were linked to the fine-mapped eQTLs.

Blinding

Because the samples were de-identified to begin with, no blinding is needed. Investigators were blinded to allocation during experiments and outcome assessments.

Reporting for specific materials, systems and methods

We require information from authors about some types of materials, experimental systems and methods used in many studies. Here, indicate whether each material, system or method listed is relevant to your study. If you are not sure if a list item applies to your research, read the appropriate section before selecting a response.

Materials & experimental systems

n/a	Involved in the study	
☒	☐	Antibodies
☒	☐	Eukaryotic cell lines
☒	☐	Palaeontology and archaeology
☒	☐	Animals and other organisms
☒	☐	Clinical data
☒	☐	Dual use research of concern

Methods

n/a	Involved in the study	
☒	☐	ChIP-seq
☒	☐	Flow cytometry
☒	☐	MRI-based neuroimaging
